# History of Diversification and Adaptation from North to South Revealed by Genomic Data: Guanacos from the Desert to Sub-Antarctica

**DOI:** 10.1093/gbe/evae085

**Published:** 2024-05-18

**Authors:** Fabiola León, Eduardo J Pizarro, Daly Noll, Luis R Pertierra, Benito A Gonzalez, Warren E Johnson, Juan Carlos Marín, Juliana A Vianna

**Affiliations:** Pontificia Universidad Católica de Chile, Facultad de Ciencias Biológicas, Instituto para el Desarrollo Sustentable, Santiago, Chile; Millennium Institute Center for Genome Regulation (CRG), Santiago, Chile; Millennium Institute Biodiversity of Antarctic and Subantarctic Ecosystems (BASE), Santiago, Chile; Millennium Nucleus of Patagonian Limit of Life (LiLi), Santiago, Chile; Pontificia Universidad Católica de Chile, Facultad de Ciencias Biológicas, Instituto para el Desarrollo Sustentable, Santiago, Chile; Millennium Institute Center for Genome Regulation (CRG), Santiago, Chile; Millennium Institute Biodiversity of Antarctic and Subantarctic Ecosystems (BASE), Santiago, Chile; Millennium Nucleus of Patagonian Limit of Life (LiLi), Santiago, Chile; Pontificia Universidad Católica de Chile, Facultad de Ciencias Biológicas, Instituto para el Desarrollo Sustentable, Santiago, Chile; Millennium Institute Center for Genome Regulation (CRG), Santiago, Chile; Millennium Institute Biodiversity of Antarctic and Subantarctic Ecosystems (BASE), Santiago, Chile; Millennium Nucleus of Patagonian Limit of Life (LiLi), Santiago, Chile; Millennium Institute Biodiversity of Antarctic and Subantarctic Ecosystems (BASE), Santiago, Chile; Laboratorio de Ecología de Vida Silvestre, Facultad de Ciencias Forestales y de la Conservación de la Naturaleza, Universidad de Chile, Santigo, Chile; Loyola University Maryland, Biology Department, USA; Laboratorio de Genómica y Biodiversidad, Departamento de Ciencias Básicas, Universidad del Bio-Bío, Chillán, Chile; Pontificia Universidad Católica de Chile, Facultad de Ciencias Biológicas, Instituto para el Desarrollo Sustentable, Santiago, Chile; Millennium Institute Center for Genome Regulation (CRG), Santiago, Chile; Millennium Institute Biodiversity of Antarctic and Subantarctic Ecosystems (BASE), Santiago, Chile; Millennium Nucleus of Patagonian Limit of Life (LiLi), Santiago, Chile

**Keywords:** camelids, extreme environment, population genomics, Atacama Desert, Patagonia

## Abstract

The increased availability of quality genomic data has greatly improved the scope and resolution of our understanding of the recent evolutionary history of wild species adapted to extreme environments and their susceptibility to anthropogenic impacts. The guanaco (*Lama guanicoe*), the largest wild ungulate in South America, is a good example. The guanaco is well adapted to a wide range of habitats, including the Sechura Desert, the high Andes Mountains to the north, and the extreme temperatures and conditions of Navarino Island to the south. Guanacos also have a long history of overexploitation by humans. To assess the evolutionary impact of these challenging habitats on the genomic diversity, we analyzed 38 genomes (∼10 to 16×) throughout their extensive latitudinal distribution from the Sechura and Atacama Desert to southward into Tierra del Fuego Island. These included analyses of patterns of unique differentiation in the north and geographic region further south with admixture among *L. g. cacsilensis* and *L. g. guanicoe*. Our findings provide new insights on the divergence of the subspecies ∼800,000 yr BP and document two divergent demographic trajectories and to the initial expansion of guanaco into the more southern portions of the Atacama Desert. Patagonian guanacos have experienced contemporary reductions in effective population sizes, likely the consequence of anthropogenic impacts. The lowest levels of genetic diversity corresponded to their northern and western limits of distribution and some varying degrees of genetic differentiation. Adaptive genomic diversity was strongly linked with environmental variables and was linked with colonization toward the south followed by adaptation.

**Significance**

The history of population differentiation and colonization in a variety of environments crossing geographical barriers is intriguing, especially when extreme habitats pose unique challenges for survival. Exploring genomic data of guanacos across diverse environments, from the Sechura Desert to Tierra del Fuego, provides clear insights on the varied influences that extreme environmental conditions have had on guanaco evolution. Strong evidence of north-to-south colonization patterns is emblematic of the historical journey of this unique American ungulate. Here, we focus on the historical and geographical factors that shaped these evolutionary genetic patterns, providing a comprehensive view of the guanaco's evolutionary history. We also explore the impacts of more contemporary challenges, including the anthropogenic impacts and climate change on Patagonian guanacos and the need for continued assessments the utility and efficacy of current conservation strategies.

## Introduction

Investigating the complex mechanisms underlying population genetic structure and divergence patterns and processes across extreme environments remains a fundamental goal of evolutionary biology, landscape genetics, and conservation genetics (Manel et al. 2003; Holderegger and Wagner 2008; Waits and Sork 2010). Understanding how landscape heterogeneity favors diversification across a species's distribution is crucial for assessing the role of the environment and the patterns of gene flow on the structuring and divergence of populations. Although factors like limited dispersal and gene flow are central to understanding population divergence and speciation (Gavrilets 2004), the influence of heterogeneous and extreme environments on the distribution of genetic diversity is fundamental, even for species of high mobility and great potential for gene flow. Identifying the key environmental determinants contributing to geographical isolation is crucial for a nuanced understanding of the dynamics and results of gene flow (Sork et al. 2010), especially for biodiversity conservation in the current context of global environmental change.

Past environmental conditions typically have a lasting impact on current genetic variability, resulting in a time lag in the response to changes in gene flow (Wright 1943; Nei and Chakravarti 1977). In particular, for species with substantial effective population sizes and extended generation times, the genetic signal of historical gene flow persists, providing accurate insights into current patterns of genetic structure (Waples 1998; Carnaval et al. 2009; Anderson et al. 2010). A detailed understanding of past macroclimatic conditions can identify and elucidate the patterns of enduring environmental changes that have discernible impacts on diversification mechanisms and the genetic diversity of species (Carnaval et al. 2009; Rodríguez-Robles et al. 2010).

The southern cone of South America exhibits a rich variety of environments, encompassing various extreme bioclimatic conditions such as the scorching heat and aridity of the Sechura and Atacama Desert, the high oxygen deprivation at high elevations in the Andes Mountains, and the cold and windy sub-Antarctic conditions in the south. One of the species that lives in this kind of ecosystem is guanaco (*Lama guanicoe*; Müller 1776), which is regarded as one of the most ecologically important and abundant native ungulate of the arid cold-temperate regions of South America, inhabiting diverse habitats from sea level to over 4,000 m (Franklin 1982; Wheeler 1995). On the western slopes of the Andes, guanacos are widely distributed from northern Peru to Tierra del Fuego and Navarino Islands in Chile (Iranzo et al. 2022). To the east, they extend southward from the Bolivian and Paraguayan Chaco, and eastward across the pampas to the Atlantic, throughout most of Argentina (Torres 1985; González et al. 2006). Since European contact 500 yr BP, guanaco populations have decreased by 95% and currently occupy only 30% of their previous range (Raedeke 1979; González et al. 2006). Overexploitation through hunting, together with habitat reduction and deterioration, and displacement by introduced livestock have caused local and regional extinctions, population reductions, and fragmentation (Torres 1992; Iranzo et al. 2013; Baldi et al. 2016).

Currently, two subspecies are recognized, *Lama g. cacsilensis* and *L. g. guanicoe*, based on analyses of sequence variation in two mitochondrial genes (Marín et al. 2008) and several microsatellite markers (Marín et al. 2013). These analyses support the notion that these two groups should be considered to be evolutionary significant units. The two subspecies diverged and became recognizably unique due to the segregation of the Andes Plateau. In the northwestern region, *L. g. cacsilensis* inhabits xeric shrub and desert ecosystems from the Pacific Ocean coast to the Puna region. In the southeast region, *L. g. guanicoe* extends through the Andes slopes to the Atlantic Ocean across Patagonian steppes, reaching its southern end on Tierra del Fuego and Navarino islands. Molecular studies of mitochondrial and microsatellite markers provide evidence of hybridization zones in the Chaco region, the Andes of central Chile and Argentina, and the western Patagonian steppe (Marín et al. 2013). Similarly, microsatellite analyses differentiated three northwestern and four to five southeastern populations, suggesting the occurrence of periodic genetic contact among these demographically independent populations, identifying possible genetic refuges and historic and more recent source-sink patterns of gene flow (Marín et al. 2013). However, these populations are not completely isolated, exemplified by evidence of extensive admixture throughout the limited contact zone and a strong signal of expansion from north to south at the beginning of Holocene (Marín et al. 2013). Despite this evolutionary perspective, the current status of the guanaco presents a contrasting scenario. The impact of human activities on genetic diversity and the recent demographic history of the species have not been explored at the local level for a few populations (González et al. 2014; Mesas, Baldi, et al. 2021; Mesas, Cuéllar-Soto, et al. 2021).

Here, we employ a genomic approach to perform a comprehensive assessment of the genetic diversity, and population structure, to infer the effect of paleoclimate and anthropogenic impact on the demographic history, and the effects of the extreme environment on the neutral and adaptive diversity of guanaco based on samples collected throughout its distribution. These results provide extensive genomic resources of guanaco and greatly enhance our understanding of the evolutionary history of the most widely distributed ungulate in South America. Additionally, the results lay the foundations for the genomic conservation and management at a species and population scale.

## Results

### Guanaco Genomic Data

**Fig. 1.**
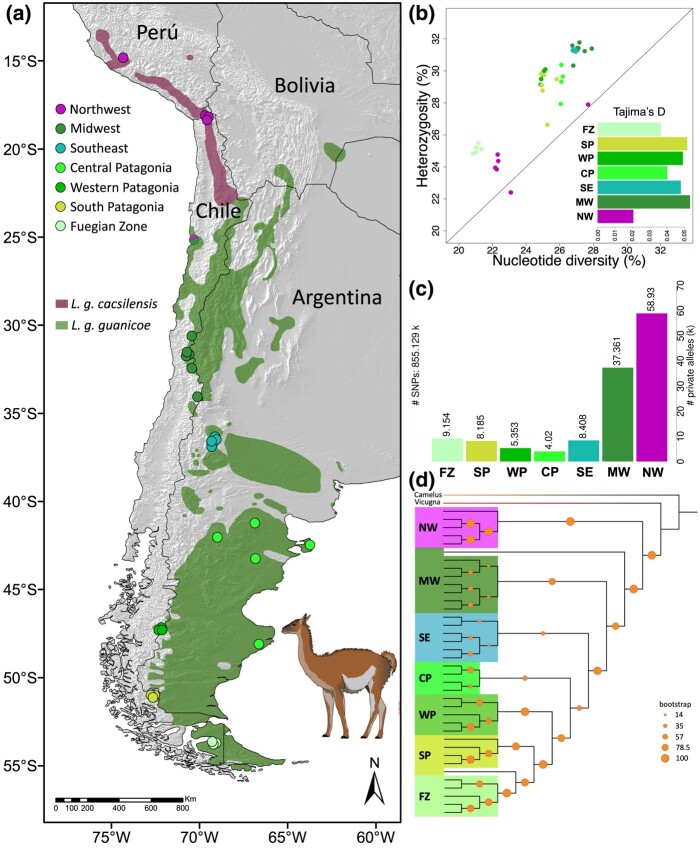
The distribution of guanacos resulting largely from genetic isolation among populations. a) A map outlining current *L. g. cacsilensis* and *L. g. guanicoe* distributions are highlighted in purple and green, respectively, along with sampled location and in colors that distinguish genetic groups (NW, northwest; MW, midwest; SE, southeast; CP, central Patagonia; WP, western Patagonia; SP, south Patagonia; FZ, Fuegian zone) identified in the phylogeny (d) and PCA (Fig. 2a). b) Nucleotide diversity and Watterson's theta of whole genomes, percentage of heterozygosity and nucleotide diversity, and the graph Tajima's *D* of each genetic group inserted. c) Graph of private alleles. d) Maximum likelihood topology generated with 1394 UCEs.

We analyzed whole-genome short-read resequencing data from 38 individuals along their latitudinal gradient distribution of the two guanaco subspecies. These genomes averaged ∼13× coverage, ranging from 10 to 16×, for the 38 genomes of *L. guanicoe* (Fig. 1a; supplementary table S1, Supplementary Material online), with a total of 11,765.6 million reads sequenced. Sexual chromosomes were removed from all the analyses. After filtering by quality, depth, minor allele frequency (MAF) < 0.05, and linkage disequilibrium (LD), 855,129 single nucleotide polymorphisms (SNPs) distributed in 36 chromosomes were retained for structure and phylogenetic analyses.

### Evolutionary History from North to South

Phylogenetic reconstruction of 1394 ultraconserved elements (UCEs) using maximum likelihood (ML) approaches revealed seven clades, branching from the northernmost location, northwest (NW), followed by midwest (MW), southeast (SE), central Patagonia (CP), western Patagonia (WP), and south Patagonia (SP) to the southernmost location Fuegian zone (FZ) of the guanaco distribution (Fig. 1d). These results indicate a northern origin for the species, with subsequent dispersal southward (Fig. 1d). The division resembles the subspecies pattern observed among the northern guanaco, *L. g. cacsilensis* (*n* = 6, Huallhua, Peru and Putre, Chile) and the other six clades of the southern guanaco, *L. g. guanicoe* (*n* = 32).

Genomic diversity (heterozygosity) was lower in insular guanaco inhabitants from the FZ locality and highest in the southern guanaco from MW (Fig. 1b) in the study. MW also showed high levels of private alleles compared to low values found in FZ (Fig. 1c). NW guanacos had the lowest levels of nucleotide diversity and heterozygosity but the highest number of private alleles in comparison with all other populations (Fig. 1b and c). Tajima's *D* analyses indicated that there was a lack of rare alleles and population contractions in both subspecies (Fig. 1b).

**Fig. 2.**
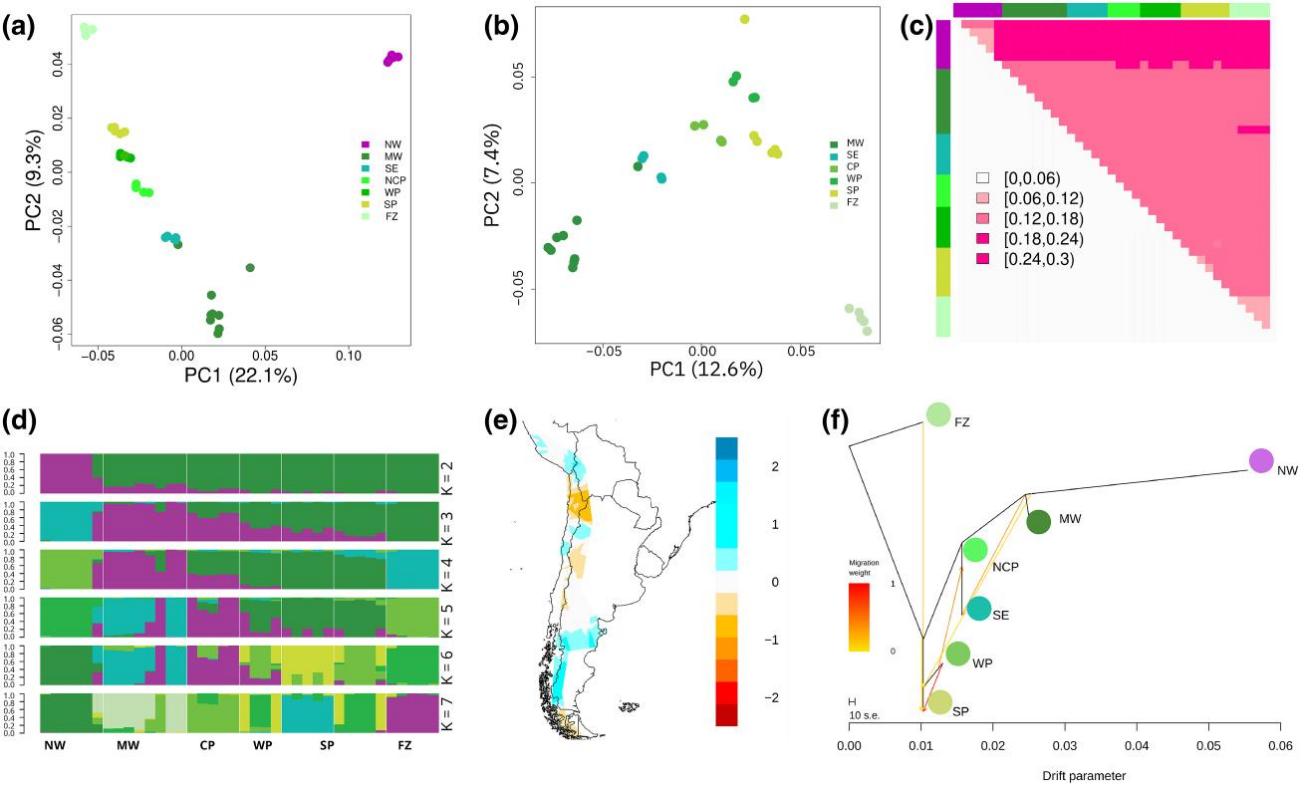
The structure of guanacos is primarily the result of the genetic isolation of natural populations. a) PCA including the sample set. b) PCA of southern guanaco. c) Nei's genetic distance matrix. d) Genetic ancestry proportion for individuals using ADMIXTURE for each of the *K* = 4 inferred ancestral populations. e) Spatial genomic structure analysis. Gene flow corridors are shown in blue and putative barriers in orange. f) TreeMix unrooted ML tree of guanaco populations with five migration edges (arrows).

Principal component analysis (PCA) of the set of filtered SNPs that encompassed all individuals discriminated three groups (Fig. 2a). The first principal component (PC1) explained 22.1% of the variance between two subspecies (*L. g. cacsilensis* and *L. g. guanicoe*), confirming that they are clearly separated. Nei genetic distance supported these subspecies results (Fig. 2c). These data differentiated individuals from Hualhuas in Peru and discriminated against the remaining northern guanacos NW in Putre, Chile. Along the second PC, the clustering pattern shows a latitudinal differentiation of *L. g. guanicoe* with 9.3% of the variance explained, being the individuals of the FZ the most differentiated group of the southern guanacos (Fig. 2a). PCA analysis of only the *L. g. guanicoe* individuals also discriminated southern guanaco MW, SE, CP, SP, and WP from insular guanaco FZ, with 12.6% of the variance explained on PC1 and 7.4% on PC2 (Fig. 2b).

The patterns of polymorphic sites in *L. g. guanicoe*, particularly in relation to the MAF class, reveal intriguing distinctions within the MW guanaco population compared to their counterparts (supplementary fig. S1, Supplementary Material online). Notably, the MW population stands out by manifesting elevated values of polymorphic sites (33%) in the lowest MAF class of 0.05, concurrently exhibiting the highest levels of segregating sites (30%). In stark contrast, guanacos from SP present a distinctive profile, showcasing the highest values of polymorphic sites (∼37%) at a notably higher frequency of 0.45, complemented by a percentage of segregating sites (20%) (supplementary fig. S1a and b, Supplementary Material online). Furthermore, individuals from *L. g. cacsilensis* in the north demonstrate the widest SNP dissimilarity among populations when compared to any other cluster delimited for *L. g guanicoe* (supplementary fig. S2, Supplementary Material online).

Admixture analyses delineated four distinct clusters (*K* = 4, Fig. 2; supplementary fig. S3, Supplementary Material online), highlighting a clear differentiation between the northernmost locations (NW) and the southernmost locations (FZ), with a gradual gradient of divergence observed in the central area, indicative of isolation by distance (Fig. 2d). The Neighbor Joining tree further reinforced this pattern, illustrating the highest divergence in the NW, followed by lower differentiation of the other groups but with higher number of differences in FZ (supplementary fig. S4, Supplementary Material online). The second most probable *K* for admixture identified two groups, differentiating the northernmost locations (NW) from others, generally described as a different subspecies (Fig. 2d). Spatial genetic structure analysis identified a region of gene flow restriction between (i) Northern guanaco and southern guanaco, (ii) southern guanaco of west and east Andes, and (iii) southern guanaco of continental and insular distribution (Fig. 2e). EEMS identifies these regions marked by distinct natural geographical barriers that influence genetic connectivity, consequently leading to restrictions in gene flow. These barriers include the arid expanses of the Sechura and Atacama Desert in northern Chile, the rugged terrain of the Andes Mountains, and the maritime barrier dividing the southern reaches of the South American continent from the expansive landscape of Tierra del Fuego. Examination of the unrooted tree, constructed via TreeMix analyses, elucidated a distinct pattern where the Northwestern Pacific population had the highest drift parameter index relative to the other subspecies (Fig. 2f). The remaining populations were tightly clustered, indicating historical and bidirectional gene flow among them. Among the southern populations—WP, SP, and FZ—a shared recent common ancestor was discernible, with the southernmost population FZ manifesting distinctive evolutionary trajectories distinct from the others (Fig. 2f). Pairwise Fst values predominantly range from 0.03 to 0.11, with elevated values specifically involving the NW in contrast to other locations (Fst = 0.07 to 0.11, supplementary fig. S5, Supplementary Material online).

### Demographic History

**Fig. 3.**
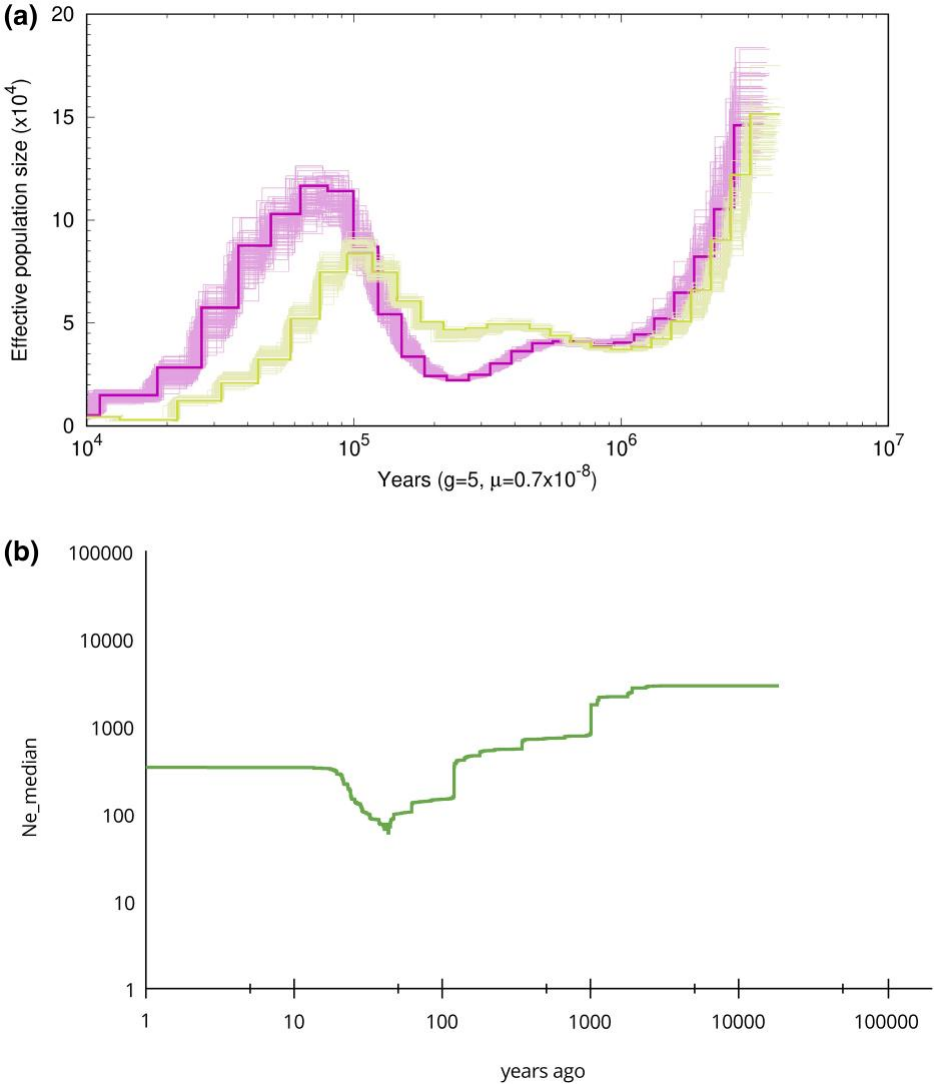
Coalescent-based inference of the demographic history of the guanaco population. a) PSMC showing two different main trajectories *L. g. cacsilensis* and *L. g. guanicoe* guanaco subspecies, purple for *L. g. cacsilensis* from NW and green for *L. g. guanicoe* from SP. b) Stairway Plot of *Lama g. guanicoe* from CP, WP, SP, and FZ.

We inferred historical changes in effective population size (Ne) using the pairwise sequentially Markovian coalescent (PSMC) model. Two different historic demographic patterns of biodiversity were observed from PSMC (Fig. 3a; supplementary fig. S6, Supplementary Material online). The two patterns between northern and southern locations, that corresponded with the two subspecies, diverged an estimated 800,000 yr BP (Fig. 3a). Our analysis revealed compelling evidence of a strong demographic expansion after ∼200,000 yr BP for southern guanacos and their northern counterparts. Interestingly, for northern guanacos, there was a population peak with a higher effective population size (Ne) around 70,000 BP, followed by a contraction. All southern guanacos showed evidence of a subsequent strong decline after ∼100,000 to ∼22,000 yr BP, overlapping with the last glacial period. The analysis of contemporary demographic history using stairway plots shows a progressive decrease in the populations of *L. g. guanicoe*, characterized by bottlenecks occurring ∼1,100 yr BP and ∼200 yr BP, with the most recent one occurring less than 100 yr BP (Fig. 3b).

Estimates of effective migration surfaces for *L. guanicoe* inferred higher than expected levels of effective migration under a model of isolation by resistance (IBR) between NW and MW, but reduced migration between MW and CP-WP-SP (Fig. 2e). Congruently, the IBR analysis showed that the structure observed in the whole population of *L. guanicoe* could be primarily steered by geographic factors and local adaptation, supporting the presence of genetic barriers in the center and northern region of its distribution.

### Genotype Environment Analysis

The constrained ordinations of genomic variation linked to the conditions of the sites surveyed allowed us to identify covarying allele frequencies in response to the extreme environmental conditions that guanacos inhabit in South American landscapes. The results of the redundancy analysis (RDA) reveal a statistically significant association of a set of environmental drivers in explaining much of the genetic variation (supplementary tables S2 to S9, Supplementary Material online) and thus provide insights into the role of the environment in shaping the adaptive genetic variation of guanaco.

**Fig. 4.**
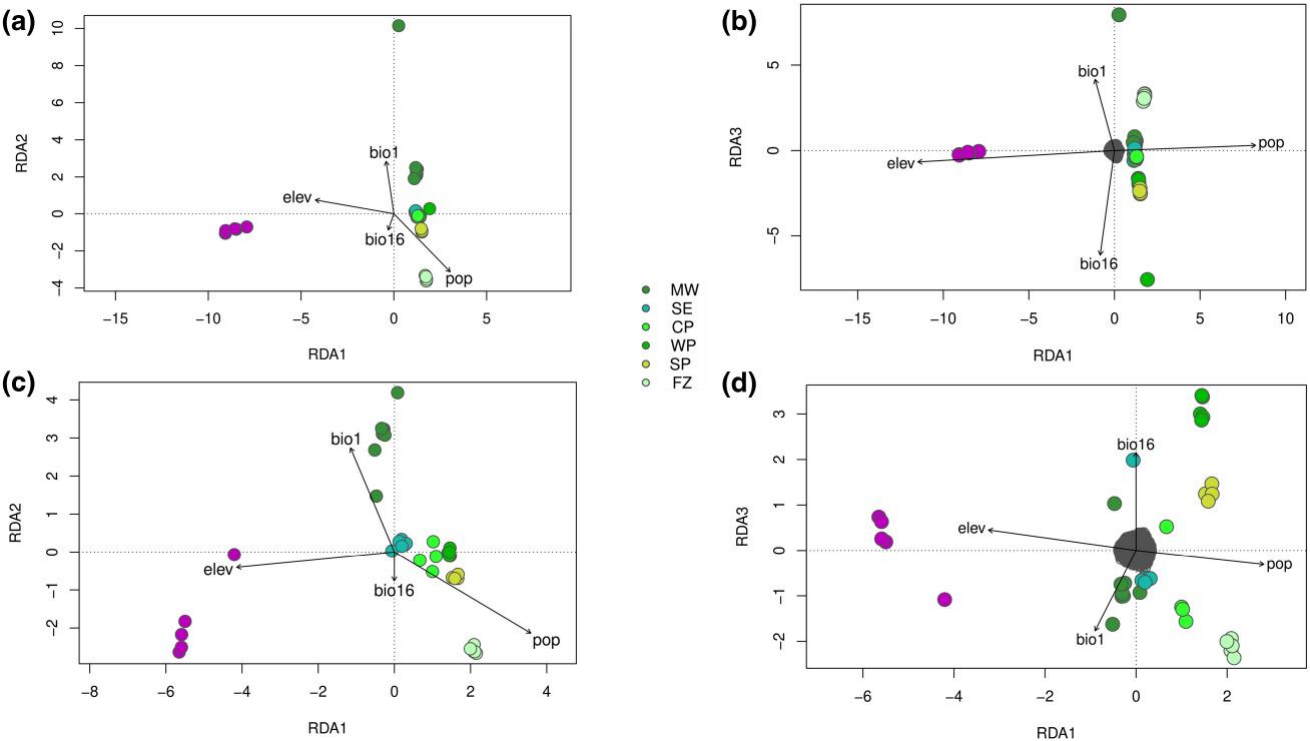
Analysis of SNPs under adaptive and neutral evolution from the guanaco metapopulation. Three significant RDA axes (*P* < 0.05) from the guanaco metapopulation adaptive data (a, b) and neutral data (c, d). This visualization encompasses colored circles that represent samples from the different population (colored circles), each potentially indicating traits or groupings, and black arrows denote signify environmental variables. RDAs provide a thorough understanding of the relationships among genomic markers, individual characteristics, and environmental influences among subspecies and across the guanaco population.

The *R*^2^ value, representing the proportion of variance in the dependent variable explained by the independent variables, is the highest (0.4909) for the adaptive set of 7,621 SNPs distributed along CDS, mRNA, exon among others (supplementary table S10, Supplementary Material online). Also, the adjusted *R*^2^, which accounts for the number of predictors providing a more conservative estimate, arouses a value of 0.4272. These estimates suggest a very strong and highly significant (*F*-statistic = 9.5659, *P* = 0.001) association between the adaptive genetic variation and the environment. It is noteworthy that RDA1, RDA2, and RDA3 maintain the association of the environmental variables with each locality (Fig. 4a and b). Specifically, individuals from the NW population of the Andean Plateau exhibit a positive correlation with elevation (ELEV) and negative correlation to population structure (POP, Fig. 4a and b). Individuals from MW, CP, WP, and SP seemed have a positive correlation with annual mean temperature (BIO1) and a negative correlation with precipitation of wettest quarter (BIO16) (Fig. 4a and b), aligned with the prevalent latitudinal-scale climate patterns in these respective regions (Fig. 4a and b).

Although both the RDA analyses for adaptive and neutral loci reveal similar clustering patterns and correlations with environmental variables, the predictive power is notably stronger for adaptive SNPs compared to neutral SNPs. Specifically, the RDA conducted using 7,265 neutral SNPs across the entire genome (supplementary tables S3 to S5, Supplementary Material online; Fig. 4c and d) indicates lower *R*^2^ values (*R*^2^ = 0.2441, adjusted *R*^2^ = 0.1236) alongside a significant *F*-statistic (*F* = 2.2701, *P* = 0.001). These findings suggest a limited association between neutral genetic variation and environmental factors. Also, it is noteworthy that, in contrast to the adaptive genetic variation, the neutral variation does not neatly correlate with the environmental pattern of the region.

In order to investigate genotype–environment associations within the southern subspecies of the guanaco (*L. g. guanicoe*), we conducted a RDA exclusively using individuals from this subspecies (supplementary figs. S7 to S9 and tables S6 to S9, Supplementary Material online). Employing Pcadapt, we identified 2,991 SNPs under selection and neutral. Individuals from the MW population of the Andean Mountains show a positive correlation with ELEV and BIO1 and a negative correlation with POP. Conversely, individuals from the WP and SP populations appear to be positively correlated with BIO16 and also with POP.

### GO and Enrichment Analysis

The gene ontology (GO) enrichment analysis conducted on 2,007 genes revealed that among the top 10 most significant GO terms, two were associated with signaling pathways, including biological processes involved in angiogenesis (GO: 0001525) and interspecies interaction between organisms (GO: 0044419), along with biological regulation (GO: 0065007). Additionally, GO terms related to the regulation of developmental (GO: 0032502) and homeostatic process (GO: 0042592). Notably, terms associated with pigmentation (GO: 0043473) and response to stimulus (GO: 0050896) were also observed.

Regarding the genomic locations of the loci potentially under selection within the 2007 genes, we observed that the majority were situated within mRNA regions, with a smaller portion (450) located in coding DNA sequences (CDS). Noteworthy findings included the identification of candidate genes associated with various known functions, such as the mechanical stability of red cells (RHCE, HS6ST1, GYPC, and FRRS1), regulation of the TOR signaling pathway (TIPRL and RHEB), melanogenesis (LRMDA), fatty acid regulation (FABP2 and FAAH2), maintenance of reactive oxygen species (ROS) homeostasis (TXNDC11 and TXNDC15), and regulation of apoptosis (CCAR2, ZYG11B, ZYG11A, and CYREN).

Our Kyoto Encyclopedia of Gene and Genomes (KEGG) analysis (supplementary fig. S10, Supplementary Material online) revealed several significant pathways associated with the population genomic variation found in this study. Pathways related to fundamental cellular processes such as axon guidance, Ras signaling, and calcium signaling were highly enriched, as evidenced by their low enrichment false discovery rate (FDR) values and substantial fold enrichments. Additionally, pathways involved in critical physiological functions like parathyroid hormone synthesis and secretion, phospholipase D signaling, and oxytocin signaling were prominently represented.

## Discussion

Our data and analyses provided details and through insights into the demography and population structure of an iconic camelid species in South America. The two lineages, confirmed by genomic analyses, show slightly different demographic histories but with population sizes decreasing toward the present. The population structure shows a clear spatial pattern of four groups with derived populations, such as NW and FZ and others that are highly connected with a gradual gradient of divergence indicative of isolation by distance. Population genomes exhibit genetic variations are linked with environmental factors such as altitude, temperature, and precipitation.

The northern hyper-arid environments exhibit more dynamic environmental gradient, with distinct historical periods that often differed from more recent patterns. Ritter et al. (2018) described paleohydrological evidence revealing the existence of Atacama Lacustrine Periods from the Pliocene to the Pleistocene. The cyclical alternation between wet and dry periods (de Porras et al. 2017) may play a crucial role in influencing variations in vegetation biomass. This, in turn, probably strengthened the availability of food resources, potentially fostering a conducive environment for the expansion of regional guanaco populations through an increased food supply.

However, the subsequent transition to extreme and drier conditions, exacerbated by the Last Glacial Maximum, introduced a multifaceted impact on the region (Latorre et al. 2002). Firstly, it reinforced the local selective establishment to hyper-arid ecoregions. Secondly, it acted as a barrier, restricting gene flow between guanaco populations and their ancestral counterparts. Thirdly, it promoted genetic divergence among populations. Lastly, the Last Glacial Maximum exerted a substantial influence on population sizes, further shaping the demographic landscape of Guanaco.

Our study supports the extended differentiation observed in the southernmost location, Tierra del Fuego Island, suggesting the increased isolation of this local population from the mainland. Evidence from guanaco bones indicates that Beagle Channel guanacos first crossed into Tierra del Fuego and Navarino Island around the time of the earliest human settlements during the Holocene, around 6,000 yr BP (Tivoli and Zangrando 2011). This leads to the notion that historical environmental shifts have had a profound influence on the genetic and ecological dynamics of guanaco populations in the region after colonization.

### Demographic History

The evolutionary trajectory of the guanaco in the desert unfolds through a divergence into two recognized subspecies around 800,000 yr ago, as revealed by Admixture, PCA, PSMC, ML phylogeny, and TreeMix analyses. The colonization of the southern cone of South America reinforced this divergence, but mantaining an intermediate geographic region of admixture. This guanaco population expansion from the north to the south is supported by paleontological, zooarchaeological, and genetic evidence using few molecular markers (Marín et al. 2013). The earliest fossils are dated from Pliocene and Late Pleistocene deposits (Scherer 2009). This leads us to postulate that the adaptive capacity of the species to the extreme environmental conditions of the Sechura and Atacama Desert and high Andes has been a persistent trait of the species since the origin of diversification.

The heightened dissimilarity in both diversity and demographic history within NW population can be ascribed to the notable variation in microenvironments along the mountain chain in the Altiplano South America's Andean Region. This geographic expanse is characterized by a variety of ecological niches that were influenced by the topological diversity of the region (Kessler 2000). The intricate mosaic of microenvironments along the mountain chain likely imposes distinct selective pressures, fostering genetic divergence among populations residing in these habitats (McCain and Grytnes 2010). Consequently, the genetic dissimilarity, as reflected by SNPs, is notably heightened in the NW, underlining the impact of the intricate mountainous terrain on shaping the genomic landscape of local populations. Consistently, high diversification in this region at the Sechura and Atacama Desert has also been suggested to other vertebrates such as Andean fox (Pizarro et al. 2024), micromammals (Palma et al. 2005), and birds (Álvarez-Varas et al. 2015).

The MW population, characterized by the lowest Tajima's *D* value and a high proportion of private alleles, suggests a history of population expansion and genetic divergence, with restricted gene flow compared to the NW population. These findings indicate a complex interplay of factors including admixture between subspecies, isolation by distance in high mountain areas, and selective pressures in extreme environments. Recent demographic expansion in the MW population, likely triggered by environmental shifts or founder effects, has led to increased genetic diversity within the group and reduced shared diversity with others. However, due to the limited sample size of MW, the full extent of recent demographic history could not be determined using the Stairway plot approach.

Within the subspecies *L. g. guanicoe*, the intricate interplay of climatic shifts is evident. As the planet's temperature decreased, this subspecies experienced a prolonged bottleneck, with estimated populations reaching their nadir during the Last Glacial Maximum around 22,000 yr BP. This extended bottleneck likely contributed to a reduction in the number of private alleles, indicative of diminished population diversity. In stark contrast, northern guanaco underwent a more expansive and prolonged population expansion, characterized by a greater acquisition of private alleles. The onset of a population bottleneck in northern guanaco preceded that in the south, emphasizing distinct mechanisms, intensities, and temporal lapses of glacial and interglacial effects on effective population size (Ne).

During the Quaternary ice ages, nuanced fluctuations in the population size of *L. g. guanicoe* across South American ecoregions become apparent. The strategic dispersal of guanacos would have played a pivotal role in colonizing geographically distant regions from their center of origin, particularly in the southern areas, including WP, SP, and FZ. Simultaneously, this dispersal strategy seemingly mitigated the impact of more extreme climate change patterns. Gene flow patterns, elucidated by TreeMix, Admixture, and EEMS analyses, underscore substantial admixing among populations. This phenomenon is likely a consequence of distribution retraction toward northern ecoregions with lesser climatic disturbances, as observed in CP. The genetic introgression previously noted by Marín et al. (2013) using mitochondrial and nuclear markers aligns with the broader evidence of population dynamics and adaptability observed in the guanaco evolutionary history.

Within the contemporaneous demographic context, discernible bottlenecks among guanaco populations emerge, revealing plausible connections with the onset of South American–Spanish colonization that began in the early 16th century. This historical epoch marked a transformative period, where heightened human activities, particularly the hunting and utilization of guanacos, contributed discernibly to demographic shifts (Diaz-Maroto et al. 2021). A progressive decrease in population size became apparent, suggesting a gradual decline possibly linked to the intensified utilization of guanacos during and after the Spanish colonization (Vergara 2018).

The hunting practices implemented by South American indigenous populations during this era could have exerted additional pressure on guanaco populations, exacerbating the demographic impact (Diaz-Maroto et al. 2021; Yacobaccio 2021). The pursuit of guanacos for resources, coupled with their domestication, may have contributed to a reduction in their overall population size. Additionally, contemporary challenges, including diminishing habitats driven by conflicts over land use, cattle farming, and agricultural expansion, have become more prevalent (Goldberg et al. 2016; Pozo et al. 2021), isolating populations and reducing observed local and global population sizes of the guanaco, whose minimum abundance was recorded between the 1950s and 1970s during the 20th century (González and Acebes 2016). The confluence of these anthropogenic influences has had a variety of discernible impacts on the demographic and genetic trajectory of extant guanacos, representing our current best assessment of their modern genetic heritage.

Wild South American camelids, specifically the guanaco *(L. guanicoe*), exhibit their adaptation to challenging desert conditions and high altitudes over the past 2 million years (Baldi et al. 2016; Acebes et al. 2018). The guanaco's ecological significance and broad distribution across diverse habitats make it an ideal candidate for unraveling the interplay between climatic factors and genetic diversity.

### Genotype Environment Analysis in Challenging Habitat

Spatial distribution of both neutral and adaptive genetic variation in guanaco using RDA unravels a clear pattern of covariation with ecological variables that likely played a pivotal role in the population's diversification. The adaptive RDA axes suggest a nuanced perspective, hinting at their potential to capture variance more intricately linked to adaptive processes, following the attenuation of prominent neutral variability. The examination of these RDA axes, across both neutral and adaptive genotypes, highlights the marked macroclimatic niche singularity of subspecies *L. g. cacsilensis* and *L. g. guanicoe*. These subspecies, characterized by disruptive geographic boundary in the desert and a low genetic association in both neutral and adaptive contexts, exhibit a constrained genetic–environment correlation. This implies that the differentiation between *L. g. cacsilensis* and *L. g. guanicoe* is primarily steered by geographic factors and local adaptation, potentially influenced by inhospitable elevation and desert habitats surrounding their respective mountain ranges, aligning with the concept of IBR as demonstrated by EEMS. The findings suggest that drift and selection influenced by a small population size and restricted gene flow significantly contributed to a divergent population model, aligning with classical evolutionary frameworks (Cortázar-Chinarro et al. 2017; Seymour et al. 2019).

Given the divergence among subspecies, our study explored the relationship between environmental variables, altitude, and genetic structure within southern guanacos, specifically *L. g. guanicoe*. Our aim was to determine the influence of both neutral and adaptive characteristics in the southernmost part of South America. Using a set of adaptive SNPs, we discovered a positive correlation between elevation and annual mean temperature in the MW population, possibly explained by the environmental differences between individuals inhabiting the Andes mountain range versus those on the Pacific coast. Similar correlations were observed with neutral loci but with less variability. Additionally, southern populations such as SP and WP were positively affected by precipitation patterns, consistent with regions characterized by heavy rainfall, particularly in winter. SNPs under selection and neutral exhibit a positive association with the FZ population, potentially due to its origin from a founder effect and genetic drift resulting from geographic isolation on the large island of Tierra del Fuego.

### Local Adaptation to Extreme Environments

Our study identifies a myriad of genes and pathways within wild guanaco populations across South America, revealing associations with climate variables. These putative adaptive loci are distributed throughout the genome, indicating a microevolutionary process of segregating genetic variation across regions. Remarkably, a substantial portion of SNPs are located within regulatory loci, suggesting that local adaptation to extreme environmental climates may exhibit polygenic traits and be influenced by the regulation of gene expression, protein functionality and, ultimately, the manifestation of phenotypic traits.

As expected for traits governed by polygenic inheritance, our analysis employing outlier detection techniques uncovered numerous genetic loci associated with both environmental and phenotypic factors. Among the biological functions potentially linked to the adaptive response to the extreme environments inhabited by guanacos are those related to homeostasis, regulation of apoptosis, and response to ROS, given the selective pressure of extreme environments (Miller et al. 2010; Sachdev et al. 2021). Our RDA findings highlight a positive correlation between subspecies and elevation, potentially elucidating selective pressures associated with oxygen deprivation. These pressures are linked to processes like angiogenesis, oxytocin signaling, and the stabilization of blood cells (Hickey and Simon 2006; Pham et al. 2021; Carter and Kingsbury 2022). Moreover, the regulation of fatty acids may assume an important role in energy storage and thermoregulation (Speakman 2018), particularly evident in cold habitats like the southern region, highlands, and Andean mountains. Additionally, significant signals of selection on mTOR may be associated with phenotypical traits such as gigantism through the cellular proliferation signaling pathway, potentially contributing to variations in size (Sarbassov et al. 2005; Herrera-Álvarez et al. 2021), among subspecies. Furthermore, distinctions in coloration, ranging from darker to lighter hues in guanacos, are thought to be related to melatonin regulation.

### Conservation Implications

The work reveals the importance of populations as evolutionary units that are subject to genomic differentiation processes, as shown by the results of adaptive genetic variability related to environmental variables. The genomes provide evidence of diverse responses to climatic and geographical variables among populations. This has direct implications for the conservation of the species since it suggests that management actions should be taken at the population scale, as has been previously suggested, using neutral markers (Marín et al. 2013). Although the guanaco can phylogenetically be considered to represent two different units, taxonomically identified as subspecies, these groups are structured internally in into populations that are subjected to different environmental pressures. In this sense, given the population genomic structure, translocations of individuals should not be taken lightly. In the event that individual translocation becomes necessary, the recommendation is to relocate individuals belonging to the same genomic and population identity, as recommended by the IUCN (2013). It is preferable for these individuals to be geographically close and originate from environments as similar as possible (Kirkwood 2013). This approach will improve the adaptive certainty of translocated individuals and facilitate the establishment of self-sustaining and functional populations in the long term (Balza et al. 2023).

On the other hand, demographic history shows a constant decline during the last century that is associated with the strong population reduction due to human causes. The lowest level of the Ne of the population coincides with the lowest level of abundance of the species (González and Acebes 2016). That reduction could be a consequence of multiple anthropogenic factors that include overexploitation, illegal hunting, modification, and transformation of the habitat to favor the occupation and expansion of livestock ranching. Fortunately, the measures taken since the 70s and 80s of the 20th century (González et al. 2022) have increased the observed size to more than 2 million animals at the present.

Our results provide salient indications of genetic differentiation within the guanaco population of Tierra del Fuego, presenting a compelling argument for its potential categorization as an evolutionarily independent unit meriting prioritized conservation consideration. This discernment underscores the overarching significance of discerning and preserving the unique genetic attributes manifested by guanacos within this specific geographic locale. The acknowledgment of the evolutionarily independent status of the Tierra del Fuego guanaco population not only constitutes a scholarly contribution but also holds pragmatic ramifications for the management and conservation perspective, since the population is under productive use (González et al. 2022) and generating conflict with sheep production and forestry (Martínez Pastur et al. 2016; Hernändez et al. 2017; Flores et al. 2023).

The conservation of distinctive genetic traits within populations has important implications for the design and execution of broader analyses of biodiversity efforts. Understanding patterns of selection among these populations and species will elucidate adaptive responses to localized environmental conditions and historical influences across broad region. The conscious recognition and protection of such evolutionarily distinct entities align with contemporary conservation paradigms, advocating for the preservation not only of species but also the intricate genetic diversity underpinning their adaptive potential found at the population level. Through these efforts, we augment our capability to sustain resilient and ecologically functional populations, fortifying their endurance amidst environmental challenges and advancing the enduring viability, in this case of the guanaco populations, and their associated ecosystems.

## Materials and Methods

### Sampling

Blood and tissue samples were collected from 38 *L. guanicoe* from 16 locations distributed along a wide latitudinal gradient from 14.65°S to 53.73°S encompassing portions of Peru, Chile, and Argentina (supplementary table S1, Supplementary Material online). DNA samples were extracted from blood and tissue samples. Blood was obtained from adults caught in the wild following chemical immobilization (darting) at ten localities, while muscle or skin samples were collected from five dead animals from five localities and five samples were obtained from five guanaco's liver slaughtered in Valle Chacabuco, Chile, under a sustainable use program authorized by the Chilean government. The wild guanacos were darted with 10 mg medetomidine and 300 mg ketamine and reversed with atipamezole per animal (modified from Georoff et al. 2010). Blood was collected in 6-ml vacutainers with EDTA that were refrigerated at −20 °C. All fieldwork was done under the supervision of a veterinarian, following the recommendations of the American Society of Mammalogists (Sikes et al. 2011).

### Genome Sequencing

DNA was extracted by DNeasy Blood & Tissue Kit (Qiagen), using a modified technique of Lahiri and Nurnberger (1991). Genomes were sequenced on an Illumina HiSeq × Ten and paired-end reads libraries were built in short inserts with an average depth coverage of ∼13×.

The whole-genome sequencing raw data were cleaned to discard any low-quality read that had any of the following criteria: (i) reads that contain 10% or more missing data; (ii) reads aligned to the adapter that exceeded in ten or more nucleotides in length; (iii) reads that aligned to the adapter with 10% or more of mismatches; (iv) reads with 50% or more of the nucleotides with phred quality score below 5; and (v) putative PCR duplicates generated during library construction.

### Quality Control and Trimming

The quality of the raw DNA sequences was assessed with FastQC v0.11.4 (Andrews 2010) and plotted with MultiQC v1.9 (Ewels et al. 2016). Raw data generated range from 35 and 45 Gb per individual. Raw reads were filtered using fastp (Chen et al. 2018), flags used include *Q* < 20, min read length of 50, and the discard of reads with Ns.

### Variant Calling

Sequenced reads were aligned to *Camelus dromedarius* (RefSeq CamDro3, accession number GCF_000803125.2) using BWA v0.7.17-r1188 (Li and Durbin 2010) with the BWA-MEM algorithm. Even though other closer species reference genomes are available such as vicuña (GCF_000164845.4) and Lama (GCA_028534125.1), they were deemed unsuitable due to the absence of GFF annotation files. Although GCA_013239585.1 had a GFF annotation file, the sheer number of resulting contigs exceeded 53,000. For this reason, we chose the reference genome from *C. dromedarius* due to its higher completeness and a more accurate gene annotation (supplementary table S11, Supplementary Material online). Alignment filtering was performed with SAMtools v1.9 (Danecek et al. 2021), using the function *samtools view* to keep only the properly paired reads (bitwise flag 0 × 2 of the SAM format) and any read with alignment quality (MAPQ) equal or higher than 10. Duplicate reads were removed with Picard MarkDuplicates v2.4.1 (Picard Toolkit. 2019. Broad Institute, GitHub Repository; http://broadinstitute.github.io/picard/; Broad Institute). Local realignment around indels was performed using RealignerTargetCreator followed by IndelRealigner from GATK v3.8 (McKenna et al. 2010). BCFtools v1.12 (Danecek et al. 2021) mpileup and call function were used to identify SNPs in each sample. Once the VCF file was obtained, it underwent filtering based on several fundamental quality parameters: SNPs located at sites covered by fewer than four reads, with an average genome coverage lower than 7.5×, and all missing data were filtered out using VCFtools v0.1.16 (Danecek et al. 2011). Additionally, SNPs found within 5 bp of an indel, and clusters of SNPs separated by less than 5 bp, were also removed from the VCF file.

### Population Structure Analysis and Population Evolutionary History

Population structure analyses were performed after selecting only biallelic SNPs and excluding singletons from the data set with BCFtools. Subsequently, to address LD, an LD-based filtering approach was applied using PLINK's (Purcell et al. 2007) “indep-pairwise” function with parameters (--indep-pairwise 50 5 0.1). This process pruned SNPs that were in high LD with each other, ensuring their independence and reducing redundancy in the analysis. Finally, to prioritize SNPs with sufficient allele variation, those with a MAF below 5% were filtered out. PCA was performed using PLINK and plotted in R. The population structure and the estimation of individual genetic ancestry to various numbers of ancestral populations (*K*) were performed using ADMIXTURE v1.3.0 (Alexander and Lange 2011). The most likely number of ancestral populations (*K*) was determined using the cross-validation error rate. Genetic differentiation among populations was estimated with Weir and Cockerham's estimator of the FST index using VCFtools. The occurrence of admixture was further investigated using TreeMix v1.12 (Pickrell and Pritchard 2012) in order to investigate the historical population relationships by estimating the ML population tree, the amount of genetic drift in each population, and the number of migration events that best fitted the data. The number of migration events (*m*) that best fitted the data was calculated by running TreeMix 15 times for each m value, with *m* ranging from 1 to 12. The optimal *m* value (*m* = 5) was estimated using the OptM R package (https://cran.r-project.org/web/packages/OptM/index.html).

### Estimation of Effective Migration Surface

To observe the degree of spatial structuring among guanaco populations and to compare the effects of landscape features on genetic variation, we analyzed patterns of gene in a spatial context using EEMS (Petkova et al. 2016), relating effective migration rates to expected genetic dissimilarities. For the latter, a biallelic matrix of genotypes was generated with PLINK and then transformed with bed2diff (https://github.com/dipetkov/eems/tree/master/bed2diffs) into a genetic differentiation matrix. The georeference of each sample was used to build a habitat polygon using http://www.birdtheme.org/useful/v3tool.html.

EEMS approach works under a stepping-stone model in which each deme exchanges migrants with only its neighbors, and the expected genetic dissimilarities depend on sample location and migration rates. EEMS was run independently for each species using the runeems_snps program using default settings; 10 million steps with one million steps burned using 400 demes. The number of sites considered varied by species (46,228 to 222,649 sites). The results were plotted with the R script reemsplots2 (https://github.com/dipetkov/reemsplots2).

### Demographic History

PSMC version 0.6.5-r67 (Li and Durbin 2011) was used to estimate the effective population size in the past. Reads alignments of each sample were individually used for variant calling using SAMtools v1.3.1, BCFtools v1.3.1, HTSlib v1.3.1, and the vcfutils.pl script with the command “samtools mpileup -C50 -uf ref.fa aln.bam | bcftools view -c - | vcfutils.pl vcf2fq -d 7.5 -D 30 gzip; diploid.fq.gz”, with -d and -D values defined according to the recommendations of the program documentation (https://github.com/lh3/psmc). The fastq file was converted to psmcfa with fq2psmcfa command, then the PSMC analysis was run with the recommended parameters “-N25 -t15 -r5 -p 4 + 25*2 + 4 + 6”. Prior to the 100 rounds of bootstraps estimates, chromosomes were fragmented with splitfa function. For the analysis, we assumed a nucleotide substitution rate of 0.7 × 10^−8^ substitutions/site/year and generation time of 5 yr based on the estimated values for South American Camelids according to Fan et al. (2020).

Only individuals with similar demographic histories, phylogeographically related, with low structure and few heterozygosity differences (among them were CP, WP, and SP) were chosen to obtain the frequency spectrum and demographic reconstruction through the Stairway plot. In this particular case, the folded site frequency spectrum (SFS) was performed because the genomic characters were not polarized with respect to an external group. The folded SFS was generated with ANGSD realSFS (Korneliussen et al. 2014), dosaf 1 -baq 1 -C 50 -minMapQ 30 -minQ 20 -P 20, to estimate demographic history through Stairway Plot v2.0 (Liu and Fu 2020), that take into account likelihood estimates to find the values that best reproduce the observed SFS and that would best estimate Ne changes through time. Mutation rate and generation time values were the same as defined for PSMC.

Demographic reconstruction analyses based on coalescence provide reliable approximations when working with genomes of moderate coverage, typically greater than 10×, and when considering factors such as duplicate read filtering, exclusion of coding sites, and sex chromosomes. The primary advantage of the PSMC approach is its ability to reconstruct both ancestral and historical demographies and, especially when dealing with a limited number of individuals per population while frequency spectrum-based demographic reconstruction offers greater confidentiality, particularly in contemporary times. In this context, we posit that both analyses serve as complementary tools, wherein the Stairway plot method fills the contemporary resolution gap inherent in PSMC, thus justifying their inclusion in this study.

### Phylogenomic Inference

The VCF obtained from the variant calling was filtered, maintaining SNPs at sites covered by four or more reads, with an average genome coverage higher than 7.5×, and missing data lower than 5% (VCFtools), while SNPs found in the vicinity of 5 bp from an indel, and clusters of SNPs separated by less than 5 bp, were filtered from the VCF (BCFtools).

A genome consensus sequence was obtained for each sample based on the VCF obtained and the reference genome (CamDro3) using bcftools consensus (BCFtools), and it was further filtered in order to maintain only the parts that were sequenced. For the latter, any site with more than one read was considered to be sequenced. The phylogenetic relationship between the samples was inferred based on the UCEs, a set of neutrally evolving whole-genome markers that capture different time span across evolution (Faircloth et al. 2012). The software PHYLUCE (Faircloth 2016) and the UCE probes for tetrapods (Faircloth et al. 2012) were used following the suggested pipeline to identify and extract the UCE from the genome consensus of each sample (Faircloth et al. 2012). The script “phyluce_probe_run_multiple_lastzs_sqlite” was used to identify the UCE in the genome, while the script “phyluce_probe_slice_sequence_from_genomes” was used to trim a flanking region of 750 bp on both sides of the UCE. The sequences were filtered following Vianna et al. (2020), keeping only sequences that had one or zero missing sites and discarding loci that were present in less than 80% of the samples. These were aligned with MAFFT v7.471 (Katoh and Standley 2013) following standard settings and then concatenated and partitioned with catfasta2phyml.pl v1.2.0 (https://github.com/nylander/catfasta2phyml).

Sequence evolutionary model was computed and selected for each partition based on the Bayesian Information Criterion with ModelTest-NG v0.1.7 (Darriba et al. 2020), which were used to perform a maximum likelihood phylogenetic inference using RAxML-NG v1.0.1 (Kozlov et al. 2019) with branch support based on Transfer Bootstrap Expectation (TBE) (Lemoine et al. 2018). The phylogeny was visualized with iTOL v5 (Letunic and Bork 2021).

### Genomic Environmental Association

We utilized RDA, a canonical ordination method developed by van den Wollenberg (1977) and Legendre and Legendre (2012), to investigate the variance in response variables explained by constraining or explanatory factors. Basic filtering criteria were applied to ensure high-quality SNP data across coding and noncoding regions, as mentioned previously in the variant calling section of our methods. Once PCAdapt identified 7,621 SNPs under selection through Bonferroni correction with *α* = 0.05 and visualized the resulting genetic variation using the first two PCs, we then selected an equivalent number of neutral SNPs using the same method. This was done to ensure that an identical count of characters was being compared in the RDA. By equalizing the number of SNPs under selection with neutral SNPs, we aimed to ensure comparability and eliminate potential biases stemming from differences in the number of characters analyzed. PCAdapt identifies neutral SNPs by reducing genomic data dimensionality, thereby facilitating the detection of loci deviating from neutral expectations. The resulting bed file for each set of SNP file was converted into an lfmm file for input into RDA approach. RDA was conducted using a systematic workflow in R, employing various packages such as vegan (Dixon 2003), LEA (Frichot and François 2015), permute (https://github.com/gavinsimpson/permute), and corrplot (Wei et al. 2017).

The climatic characteristics of each site were compared across 19 bioclimatic variables from northern and southern guanacos, which were extracted with R software packages: terra (Hijmans et al. 2022), raster (https://github.com/rspatial/raster), and rgdal (Bivand 2015) at 30 arc-second resolutions from CHELSA database (Karger et al. 2017) covering the period 1980 to 2010. The median elevation was obtained from SRTM4.1 global topography (Amatulli et al. 2018). To choose the environmental variables for the analysis, the correlation between variables was examined. Variables with *R*^2^ value under 0.77 were considered as variables with low correlation and were kept for further steps (summary data available in supplementary table S2, Supplementary Material online). Genetic and environmental data were inspected for data structure and filtered for missing values. The best subsetting of variables explored in the RDA analysis (i.e. those maximizing the *R*^2^ value while being highly significant) comprised population, elevation, BIO16, BIO17, BIO18, BIO15, BIO1, BIO2, BIO3, and BIO4 (supplementary table S2 and fig. S7, Supplementary Material online). The RDA was executed with genetic data regressed on the selected environmental variables that were previously standardized.

Due to the significant divergence between northern and southern subspecies of guanacos, we conducted an additional RDA focusing exclusively on the southern individuals from MW, SE, CP, WP, SP, and FZ localities. We employed the same workflow for identifying neutral and putative adaptive SNPs for both subspecies, as previously mentioned. This process resulted in the identification of 2,991 SNPs under selection and 777,497 under neutral. The identification of the best subset of variables in the RDA analysis, defined as those maximizing the *R*^2^ value while maintaining high significance, included POP structure, ELEVATION, BIO1, and BIO16 (refer to supplementary table S2 and fig. S7, Supplementary Material online). Subsequently, the RDA was conducted with genetic data regressed on the selected environmental variables, which were standardized beforehand.

The results were examined comprehensively using several robust analytical approaches including assessments of eigenvalues and adjusted *R*^2^ values, and significance tests were performed with 999 permutations for (i) the RDA model, (ii) the terms of the model (added sequentially), and (iii) the axes of the model (supplementary tables S6 to S9, Supplementary Material online). Visualization of the RDA outputs was exhibited through scatter plots, providing insights into the relationships between genetic and environmental factors.

### GO and Enrichment Analysis

We employed KEGG (Kanehisa 2002) and Panther (Mi et al. 2019) to predict biological functionality and interrelationships among genes. Subsequently, we generated dot plots depicting the KEGG pathway, FDR, and the number of genes involved in each pathway. FDR is calculated based on the nominal *P*-value from the hypergeometric test. Fold enrichment is defined as the percentage of genes list obtained belonging to a pathway, divided by the corresponding percentage in the background. FDR explains how likely the enrichment is by chance, and large pathways tend to have smaller FDRs, as a measure of effect size.

## Supplementary Material

evae085_Supplementary_Data

## Data Availability

Guanacos raw fastq reads have been deposited in the GenBank database (BioProject PRJNA1043467 and BioSample accession numbers SRR26902306 to SRR26902344). All scripts are available at GitHub (https://github.com/lafabi). All other data are provided in either the main text or the Supplementary material.

## References

[evae085-B1] Acebes P, Wheeler J, Baldo J, Tuppia P, Lichtenstein G, Hoces D, Franklin WL. *Vicugna vicugna* (errata version published in 2019). The IUCN Red List of Threatened Species 2018. 2018. 10.2305/IUCN.UK.2018-2.RLTS.T22956A145360542.en.

[evae085-B2] Alexander DH, Lange K. Enhancements to the ADMIXTURE algorithm for individual ancestry estimation. BMC Bioinformatics. 2011:12(1):246. 10.1186/1471-2105-12-246. 21682921 PMC3146885

[evae085-B3] Álvarez-Varas R, González-Acuña D, Vianna JA. Comparative phylogeography of co-distributed *Phrygilus* species (Aves, Thraupidae) from the Central Andes. Mol Phylogenet Evol. 2015:90:150–163. 10.1016/j.ympev.2015.04.009. 25987531

[evae085-B4] Amatulli G, Domisch S, Tuanmu M-N, Parmentier B, Ranipeta A, Malczyk J, Jetz W. A suite of global, cross-scale topographic variables for environmental and biodiversity modeling. Sci Data. 2018:5(1):180040. 10.1038/sdata.2018.40. 29557978 PMC5859920

[evae085-B5] Anderson CD, Epperson BK, Fortin M-J, Holderegger R, James PMA, Rosenberg MS, Scribner KT, Spear S. Considering spatial and temporal scale in landscape-genetic studies of gene flow. Mol Ecol. 2010:19(17):3565–3575. 10.1111/j.1365-294X.2010.04757.x. 20723051

[evae085-B6] Andrews S . FastQC: a quality control tool for high throughput sequence data. 2010. Available online at: http://www.bioinformatics.babraham.ac.uk/projects/fastqc/.

[evae085-B7] Baldi RPM, Acebes P, Cuéllar E, Funes M, Hoces D, Puig S, Franklin WL. Lama guanicoe. IUCN Red List Threat. Species. UK: The IUCN; 2016. 1. 10.2305/IUCN.UK.2016-1.RLTS.T11186A18540211.en.

[evae085-B8] Balza U, Baldi R, Rodríguez-Planes L, Ojeda R, Schiavini A. Scientific evidence does not support the translocation of guanacos in Argentina. Conserv Sci Pract. 2023:5(11):e13031. 10.1111/csp2.13031.

[evae085-B9] Bivand R. 2015. Package ‘rgdal’. Bind. Geospatial Data Abstr. Libr. [accessed 2017 Oct 15]. Available online at: https://cran.r-project.org/web/packages/rgdal/index.html. 172.

[evae085-B10] Carnaval AC, Hickerson MJ, Haddad CFB, Rodrigues MT, Moritz C. Stability predicts genetic diversity in the Brazilian Atlantic forest hotspot. Science. 2009:323(5915):785–789. 10.1126/science.1166955. 19197066

[evae085-B11] Carter CS, Kingsbury MA. Oxytocin and oxygen: the evolution of a solution to the ‘stress of life’. Philos Trans R Soc Lond B Biol Sci. 2022:377(1858):20210054. 10.1098/rstb.2021.0054. 35856299 PMC9272143

[evae085-B12] Chen S, Zhou Y, Chen Y, Gu J. Fastp: an ultra-fast all-in-one FASTQ preprocessor. Bioinformatics. 2018:34(17):i884–i890. 10.1093/bioinformatics/bty560. 30423086 PMC6129281

[evae085-B13] Cortázar-Chinarro M, Lattenkamp EZ, Meyer-Lucht Y, Luquet E, Laurila A, Höglund J. Drift, selection, or migration? Processes affecting genetic differentiation and variation along a latitudinal gradient in an amphibian. BMC Evol Biol. 2017:17(1):189. 10.1186/s12862-017-1022-z. 28806900 PMC5557520

[evae085-B14] Danecek P, Auton A, Abecasis G, Albers CA, Banks E, DePristo MA, Handsaker RE, Lunter G, Marth GT, Sherry ST, et al The variant call format and VCFtools. Bioinformatics. 2011:27(15):2156–2158. 10.1093/bioinformatics/btr330. 21653522 PMC3137218

[evae085-B15] Danecek P, Bonfield JK, Liddle J, Marshall J, Ohan V, Pollard MO, Whitwham A, Keane T, McCarthy SA, Davies RM, et al Twelve years of SAMtools and BCFtools. GigaScience. 2021:10(2):giab008. 10.1093/gigascience/giab008. 33590861 PMC7931819

[evae085-B16] Darriba D, Posada D, Kozlov AM, Stamatakis A, Morel B, Flouri T. ModelTest-NG: a new and scalable tool for the selection of DNA and protein evolutionary models. Mol Biol Evol. 2020:37(1):291–294. 10.1093/molbev/msz189. 31432070 PMC6984357

[evae085-B17] de Porras ME, Maldonado A, De Pol-Holz R, Latorre C, Betancourt JL. Late Quaternary environmental dynamics in the Atacama Desert reconstructed from rodent midden pollen records. J Quat Sci. 2017:32(6):665–684. 10.1002/jqs.2980.

[evae085-B18] Diaz-Maroto P, Rey-Iglesia A, Cartajena I, Núñez L, Westbury MV, Varas V, Moraga M, Campos PF, Orozco-terWengel P, Marin JC, et al Ancient DNA reveals the lost domestication history of South American camelids in Northern Chile and across the Andes. eLife. 2021:10:e63390. 10.7554/eLife.63390. 33724183 PMC8032396

[evae085-B19] Dixon P . VEGAN, a package of R functions for community ecology. J Veg Sci. 2003:14(6):927–930. 10.1111/j.1654-1103.2003.tb02228.x.

[evae085-B20] Ewels P, Magnusson M, Lundin S, Käller M. MultiQC: summarize analysis results for multiple tools and samples in a single report. Bioinformatics. 2016:32(19):3047–3048. 10.1093/bioinformatics/btw354. 27312411 PMC5039924

[evae085-B21] Faircloth BC . PHYLUCE is a software package for the analysis of conserved genomic loci. Bioinformatics. 2016:32(5):786–788. 10.1093/bioinformatics/btv646. 26530724

[evae085-B22] Faircloth BC, McCormack JE, Crawford NG, Harvey MG, Brumfield RT, Glenn TC. Ultraconserved elements anchor thousands of genetic markers spanning multiple evolutionary timescales. Syst Biol. 2012:61(5):717–726. 10.1093/sysbio/sys004. 22232343

[evae085-B23] Fan R, Gu Z, Guang X, Marín JC, Varas V, González BA, Wheeler JC, Hu Y, Li E, Sun X, et al Genomic analysis of the domestication and post-Spanish conquest evolution of the llama and alpaca. Genome Biol. 2020:21(1):159. 10.1186/s13059-020-02080-6. 32616020 PMC7331169

[evae085-B24] Flores C, Lichtenstein G, Schiavini A. Human–wildlife conflicts in Patagonia: ranchers’ perceptions of guanaco *Lama guanicoe* abundance. Oryx. 2023:57(5):615–625. 10.1017/S0030605322001508.

[evae085-B25] Franklin WL . Biology, ecology, and relationship to man of the South American camelids. Mamm Biol S Am. 1982:6:457–489.

[evae085-B26] Frichot E, François O. LEA: an R package for landscape and ecological association studies. Methods Ecol Evol. 2015:6(8):925–929. 10.1111/2041-210X.12382.

[evae085-B27] Gavrilets S . Fitness landscapes and the origin of species. Princeton, NJ: Princeton University Press; 2004.

[evae085-B28] Georoff TA, James SB, Kalk P, Calle PP, Martin-Flores M. Evaluation of medetomidine–ketamine–butorphanol anesthesia with atipamezole–naltrexone antagonism in captive male guanacos (*Lama guanicoe*). J Zoo Wildl Med. 2010:41(2):255–262. 10.1638/2009-0203R.1. 20597217

[evae085-B29] Goldberg A, Mychajliw AM, Hadly EA. Post-invasion demography of prehistoric humans in South America. Nature. 2016:532(7598):232–235. 10.1038/nature17176. 27049941

[evae085-B30] González BA, Acebes P. Reevaluación del guanaco para la Lista Roja de la UICN: situación actual y recomendaciones a futuro. GECS News. 2016:6:15–21.

[evae085-B31] González BA, Acebes P, Corti P, Grimberg M, Iranzo E, Malo JE, Moraga CA, Sarno RJ, Skewes O, Soto N, et al Historical perspective and current understanding of the ecology, conservation, and management of the guanaco in the Chilean Patagonia. In: Carmanchahi P, Lichtenstein G, editors. Guanacos and people in Patagonia: a social-ecological approach to a relationship of conflicts and opportunities. Cham: Natural and Social Sciences of Patagonia Springer International Publishing; 2022. p. 191–232.

[evae085-B32] González BA, Orozco-terWengel P, von Borries R, Johnson WE, Franklin WL, Marín JC. Maintenance of genetic diversity in an introduced island population of guanacos after seven decades and two severe demographic bottlenecks: implications for camelid conservation. PLoS One. 2014:9(3):e91714. 10.1371/journal.pone.0091714. 24663026 PMC3963871

[evae085-B33] González BA, Palma RE, Zapata B, Marín JC. Taxonomic and biogeographical status of guanaco *Lama guanicoe* (Artiodactyla, Camelidae). Mammal Rev. 2006:36(2):157–178. 10.1111/j.1365-2907.2006.00084.x.

[evae085-B34] Hernändez F, Corcoran D, Graells G, Rõos C, Downey MC. Rancher perspectives of a livestock-wildlife conflict in southern Chile. Rangelands. 2017:39(2):56–63. 10.1016/j.rala.2017.02.002.

[evae085-B35] Herrera-Álvarez S, Karlsson E, Ryder OA, Lindblad-Toh K, Crawford AJ. How to make a rodent giant: genomic basis and tradeoffs of gigantism in the capybara, the world's largest rodent. Mol Biol Evol. 2021:38(5):1715–1730. 10.1093/molbev/msaa285. 33169792 PMC8097284

[evae085-B36] Hickey MM, Simon MC. Regulation of angiogenesis by hypoxia and hypoxia-inducible factors. Curr Top Dev Biol. 2006:76:217–257. 10.1016/S0070-2153(06)76007-0. 17118268

[evae085-B37] Hijmans RJ, Hijmans RJ, Bivand R, Forner K, Ooms J, Pebesma E, Sumner MD. Package ‘terra’. Vienna, Austria: Maint; 2022.

[evae085-B38] Holderegger R, Wagner HH. Landscape genetics. BioScience. 2008:58(3):199–207. 10.1641/B580306.

[evae085-B39] Iranzo EC, Smith C, Moraga CA, Radic-Schilling S, Corti P. Patterns of guanaco distribution and microhabitat use in Tierra del Fuego: from protected to sheep ranching areas. Acta Oecol. 2022:116:103853. 10.1016/j.actao.2022.103853.

[evae085-B40] Iranzo EC, Traba J, Acebes P, González BA, Mata C, Estades CF, Malo JE. Niche segregation between wild and domestic herbivores in Chilean Patagonia. PLoS One. 2013:8(3):e59326. 10.1371/journal.pone.0059326. 23555656 PMC3605456

[evae085-B41] IUCN/SSC . Guidelines for reintroductions and other conservation translocations. Version 1.0. Gland, Switzerland: IUCN Species Survival Commission; 2013. viiii + 57 pp.

[evae085-B42] Kanehisa M . The KEGG database. ‘In silico’ simulation of biological processes: Novartis Foundation Symposium 247. 247. Chichester, UK: John Wiley & Sons, Ltd; 2002. p. 91–103. 12539951

[evae085-B43] Karger DN, Conrad O, Böhner J, Kawohl T, Kreft H, Soria-Auza RW, Zimmermann NE, Linder HP, Kessler M. Climatologies at high resolution for the earth's land surface areas. Sci Data. 2017:4(1):170122. 10.1038/sdata.2017.122. 28872642 PMC5584396

[evae085-B45] Katoh K, Standley DM. MAFFT multiple sequence alignment software version 7: improvements in performance and usability. Mol Biol Evol. 2013:30(4):772–780. 10.1093/molbev/mst010. 23329690 PMC3603318

[evae085-B46] Kessler M . Elevational gradients in species richness and endemism of selected plant groups in the central Bolivian Andes. Plant Ecol. 2000:149(2):181–193. 10.1023/A:1026500710274.

[evae085-B47] Kirkwood JK . Guidelines for reintroductions and other conservation translocations. Anim Welf. 2013:22:489–490. 10.1017/S0962728600005637.

[evae085-B48] Korneliussen TS, Albrechtsen A, Nielsen R. ANGSD: analysis of next generation sequencing data. BMC Bioinformatics. 2014:15(1):356. 10.1186/s12859-014-0356-4. 25420514 PMC4248462

[evae085-B49] Kozlov AM, Darriba D, Flouri T, Morel B, Stamatakis A. RAxML-NG: a fast, scalable and user-friendly tool for maximum likelihood phylogenetic inference. Bioinformatics. 2019:35(21):4453–4455. 10.1093/bioinformatics/btz305. 31070718 PMC6821337

[evae085-B50] Lahiri DK, Nurnberger JI Jr. A rapid non-enzymatic method for the preparation of HMW DNA from blood for RFLP studies. Nucleic Acids Res. 1991:19(19):5444. 10.1093/nar/19.19.5444. 1681511 PMC328920

[evae085-B51] Latorre C, Betancourt JL, Rylander KA, Quade J. Vegetation invasions into absolute desert: a 45;th000 yr rodent midden record from the Calama–Salar de Atacama basins, northern Chile (lat 22°–24°S). Geol Soc Am Bull. 2002:114(3):349–366. 10.1130/0016-7606(2002)114<0349:VIIADA>2.0.CO;2.

[evae085-B52] Legendre P, Legendre L. Numerical ecology. 3rd ed., Vol. 24. Quebéc, Canada: Elsevier; 2012. 990 pp.

[evae085-B53] Lemoine F, Domelevo Entfellner J-B, Wilkinson E, Correia D, Dávila Felipe M, De Oliveira T, Gascuel O. Renewing Felsenstein's phylogenetic bootstrap in the era of big data. Nature. 2018:556(7702):452–456. 10.1038/s41586-018-0043-0. 29670290 PMC6030568

[evae085-B54] Letunic I, Bork P. Interactive Tree Of Life (iTOL) v5: an online tool for phylogenetic tree display and annotation. Nucleic Acids Res. 2021:49(W1):W293–W296. 10.1093/nar/gkab301. 33885785 PMC8265157

[evae085-B55] Li H, Durbin R. Fast and accurate long-read alignment with Burrows–Wheeler transform. Bioinformatics. 2010:26:589–595. 20080505 10.1093/bioinformatics/btp698 PMC2828108

[evae085-B56] Li H, Durbin R. Inference of human population history from individual whole-genome sequences. Nature. 2011:475(7357):493–496. 10.1038/nature10231. 21753753 PMC3154645

[evae085-B57] Liu X, Fu Y-X. Stairway Plot 2: demographic history inference with folded SNP frequency spectra. Genome Biol. 2020:21(1):280. 10.1186/s13059-020-02196-9. 33203475 PMC7670622

[evae085-B58] Manel S, Schwartz MK, Luikart G, Taberlet P. Landscape genetics: combining landscape ecology and population genetics. Trends Ecol Evol. 2003:18(4):189–197. 10.1016/S0169-5347(03)00008-9.

[evae085-B59] Marín JC, González BA, Poulin E, Casey CS, Johnson WE. The influence of the arid Andean high plateau on the phylogeography and population genetics of guanaco (*Lama guanicoe*) in South America. Mol Ecol. 2013:22(2):463–482. 10.1111/mec.12111. 23206254 PMC3549358

[evae085-B60] Marín JC, Spotorno AE, González BA, Bonacic C, Wheeler JC, Casey CS, Bruford MW, Palma RE, Poulin E. Mitochondrial DNA variation and systematics of the guanaco (*Lama guanicoe*, Artiodactyla: Camelidae). J Mammal. 2008:89(2):269–281. 10.1644/06-MAMM-A-385R.1.

[evae085-B61] Martínez Pastur G, Soler R, Ivancich H, Lencinas MV, Bahamonde H, Peri PL. Effectiveness of fencing and hunting to control *Lama guanicoe* browsing damage: implications for *Nothofagus pumilio* regeneration in harvested forests. J Environ Manage. 2016:168:165–174. 10.1016/j.jenvman.2015.11.051. 26708647

[evae085-B62] McCain CM, Grytnes J-A. Elevational gradients in species richness. Encyclopedia of Life Sciences (ELS). Chichester: John Wiley & Sons, Ltd; 2010 10.1002/9780470015902.a0022548.

[evae085-B63] McKenna A, Hanna M, Banks E, Sivachenko A, Cibulskis K, Kernytsky A, Garimella K, Altshuler D, Gabriel S, Daly M, et al The Genome Analysis Toolkit: a MapReduce framework for analyzing next-generation DNA sequencing data. Genome Res. 2010:20(9):1297–1303. 10.1101/gr.107524.110. 20644199 PMC2928508

[evae085-B64] Mesas A, Baldi R, González BA, Burgi V, Chávez A, Johnson WE, Marín JC. Past and recent effects of livestock activity on the genetic diversity and population structure of native guanaco populations of arid patagonia. Animals (Basel). 2021:11(5):1218. 10.3390/ani11051218. 33922526 PMC8146674

[evae085-B65] Mesas A, Cuéllar-Soto E, Romero K, Zegers T, Varas V, González BA, Johnson WE, Marín JC. Assessing patterns of genetic diversity and connectivity among guanacos (*Lama guanicoe*) in the Bolivian Chaco: implications for designing management strategies. Stud Neotrop Fauna Environ. 2021:58(1):94–103. 10.1080/01650521.2021.1914294.

[evae085-B66] Mi H, Muruganujan A, Ebert D, Huang X, Thomas PD. PANTHER version 14: more genomes, a new PANTHER GO-slim and improvements in enrichment analysis tools. Nucleic Acids Res. 2019:47(D1):D419–D426. 10.1093/nar/gky1038. 30407594 PMC6323939

[evae085-B67] Miller G, Suzuki N, Ciftci-Yilmaz S, Mittler R. Reactive oxygen species homeostasis and signalling during drought and salinity stresses. Plant Cell Environ. 2010:33(4):453–467. 10.1111/j.1365-3040.2009.02041.x. 19712065

[evae085-B68] Müller PLS . Erste classe, säugende thiere. Des Ritters Carl von Linné vollständiges Naturalsystem nach der zwölften Lateinischen Ausgabe. 1776:1773–1776. pp. 1–62 + 3 pls., Suppl. 384 pp, Register, 36 unnumbered pp. + 536 pp.

[evae085-B69] Nei M, Chakravarti A. Drift variances of FST and GST statistics obtained from a finite number of isolated populations. Theor Popul Biol. 1977:11(3):307–325. 10.1016/0040-5809(77)90014-4. 877909

[evae085-B70] Palma RE, Marquet PA, Boric-Bargetto D. Inter- and intraspecific phylogeography of small mammals in the Atacama Desert and adjacent areas of northern Chile. J Biogeogr. 2005:32(11):1931–1941. 10.1111/j.1365-2699.2005.01349.x.

[evae085-B71] Petkova D, Novembre J, Stephens M. Visualizing spatial population structure with estimated effective migration surfaces. Nat Genet. 2016:48(1):94–100. 10.1038/ng.3464. 26642242 PMC4696895

[evae085-B72] Pham K, Parikh K, Heinrich EC. Hypoxia and inflammation: insights from high-altitude physiology. Front Physiol. 2021:12:676782. 10.3389/fphys.2021.676782. 34122145 PMC8188852

[evae085-B73] Pickrell J, Pritchard J. Inference of population splits and mixtures from genome-wide allele frequency data. Nat Preced. 2012:1–1. 10.1038/npre.2012.6956.1. PMC3499260 23166502

[evae085-B74] Pizarro EJ, Julio-Kalajžić B, Sallaberry-Pincheira N, Muñoz V, González-Acuña D, Cabello J, Acosta-Jamett G, Bonacic C, Iriarte A, Rodríguez A, et al Species delimitation and intraspecific diversification in recently diverged South American foxes. Mammal Res. 2024:69:71–87. 10.1007/s13364-023-00717-y.

[evae085-B75] Pozo RA, Cusack JJ, Acebes P, Malo JE, Traba J, Iranzo EC, Morris-Trainor Z, Minderman J, Bunnefeld N, Radic-Schilling S, et al Reconciling livestock production and wild herbivore conservation: challenges and opportunities. Trends Ecol Evol. 2021:36(8):750–761. 10.1016/j.tree.2021.05.002. 34103191

[evae085-B76] Purcell S, Neale B, Todd-Brown K, Thomas L, Ferreira MAR, Bender D, Maller J, Sklar P, de Bakker PIW, Daly MJ, et al PLINK: a tool set for whole-genome association and population-based linkage analyses. Am J Hum Genet. 2007:81(3):559–575. 10.1086/519795. 17701901 PMC1950838

[evae085-B77] Raedeke KJ . Population dynamics and socioecology of the guanaco (Lama guanicoe) of magallanes. Chile: University of Washington; 1979.

[evae085-B78] Ritter B, Binnie SA, Stuart FM, Wennrich V, Dunai TJ. Evidence for multiple plio-pleistocene lake episodes in the hyperarid atacama desert. Quat Geochronol. 2018:44:1–12. 10.1016/j.quageo.2017.11.002.

[evae085-B79] Rodríguez-Robles JA, Jezkova T, Leal M. Climatic stability and genetic divergence in the tropical insular lizard *Anolis krugi*, the Puerto Rican ‘Lagartijo Jardinero de la Montaña’. Mol Ecol. 2010:19(9):1860–1876. 10.1111/j.1365-294X.2010.04616.x. 20374489

[evae085-B80] Sachdev S, Ansari SA, Ansari MI, Fujita M, Hasanuzzaman M. Abiotic stress and reactive oxygen species: generation, signaling, and defense mechanisms. Antioxidants (Basel). 2021:10(2):277. 10.3390/antiox10020277. 33670123 PMC7916865

[evae085-B81] Sarbassov DD, Ali SM, Sabatini DM. Growing roles for the mTOR pathway. Curr Opin Cell Biol. 2005:17(6):596–603. 10.1016/j.ceb.2005.09.009. 16226444

[evae085-B82] Scherer CS, Pitana VG, Ribeiro AM. Proterotheriidae and Macraucheniidae (Litopterna, Mammalia) from the Pleistocene of Rio Grande do Sul State, Brazil. Rev Bras Paleontol. 2009:12(3):231–246.

[evae085-B83] Seymour M, Räsänen K, Kristjánsson BK. Drift versus selection as drivers of phenotypic divergence at small spatial scales: the case of belgjarskógur threespine stickleback. Ecol Evol. 2019:9(14):8133–8145. 10.1002/ece3.5381. 31380077 PMC6662300

[evae085-B84] Sikes RS, Gannon WL; the Animal Care and Use Committee of the American Society of Mammalogists. Guidelines of the American Society of Mammalogists for the use of wild mammals in research. J Mammal. 2011:92(1):235–253. 10.1644/10-MAMM-F-355.1. PMC5909806 29692469

[evae085-B85] Sork VL, Davis FW, Westfall R, Flint A, Ikegami M, Wang H, Grivet D. Gene movement and genetic association with regional climate gradients in California valley oak (Quercus lobata Née) in the face of climate change. Mol Ecol. 2010:19(17):3806–3823. 10.1111/j.1365-294X.2010.04726.x. 20723054

[evae085-B86] Speakman JR . Obesity and thermoregulation. Handb Clin Neurol. 2018:156:431–443. 10.1016/B978-0-444-63912-7.00026-6. 30454605

[evae085-B87] Tivoli AM, Zangrando AF. Subsistence variations and landscape use among maritime hunter-gatherers. A zooarchaeological analysis from the Beagle Channel (Tierra del Fuego. Argentina). J Archaeol Sci. 2011:38(5):1148–1156. 10.1016/j.jas.2010.12.018.

[evae085-B88] Torres H . Guanaco: distribución y conservación del guanaco. IUCNCSE Grupo Espec. Lond, UK: En Camélidos Sudam; 1985.

[evae085-B89] Torres H . South American Camelids: an action plan for their conservation = camélidos silvestres sudamericanos: un plan de acción para su conservación. Gland, Switzerland: IUCN; 1992. [accessed 2023 Nov 25]. https://portals.iucn.org/library/node/6004.

[evae085-B90] van den Wollenberg AL . Redundancy analysis an alternative for canonical correlation analysis. Psychometrika. 1977:42(2):207–219. 10.1007/BF02294050.

[evae085-B91] Vergara G . Animals in latin american history. Oxford research encyclopedia of Latin American history. USA: Oxford University Press; 2018. 10.1093/acrefore/9780199366439.013.436.

[evae085-B92] Vianna JA, Fernandes FAN, Frugone MJ, Figueiró HV, Pertierra LR, Noll D, Bi K, Wang-Claypool CY, Lowther A, Parker P, et al Genome-wide analyses reveal drivers of penguin diversification. Proc Natl Acad Sci U S A. 2020:117(36):22303–22310. 10.1073/pnas.2006659117. 32817535 PMC7486704

[evae085-B93] Waits L, Sork VL. Special issue: landscape genetics. Mol Ecol. 2010:19(17):3489–3835. 10.1111/j.1365-294X.2010.04786.x. 20723050

[evae085-B94] Waples R . Separating the wheat from the chaff: patterns of genetic differentiation in high gene flow species. J Hered. 1998:89(5):438–450. 10.1093/jhered/89.5.438.

[evae085-B95] Wei T, Wei T, Simko V, Levy M, Xie Y, Jin Y, Zemla J. Package ‘corrplot’. Statistician. 2017:56:e24. https://cran.r-project.org/web/packages/corrplot/corrplot.pdf

[evae085-B96] Wheeler JC . Evolution and present situation of the South American Camelidae. Biol J Linn Soc. 1995:54(3):271–295. 10.1016/0024-4066(95)90021-7.

[evae085-B97] Wright S . Isolation by distance. Genetics. 1943:28(2):114–138. 10.1093/genetics/28.2.114. 17247074 PMC1209196

[evae085-B98] Yacobaccio HD . The domestication of South American camelids: a review. Anim Front. 2021:11(3):43–51. 10.1093/af/vfaa065. PMC8214430 34158988

