# Supplementary material for: History of Diversification and Adaptation from North to South Revealed by Genomic Data: Guanacos from the Desert to Sub-Antarctica
Source: Genome Biol Evol. 2024 May 18;16(5):evae085. doi: 10.1093/gbe/evae085 (PMC11102080; doi:10.1093/gbe/evae085)
Supplement: evae085_Supplementary_Data [file evae085_supplementary_data.zip › SupplementaryMaterial_Figures_Leon.docx]

**Supplementary Material Figures**

**History of diversification and adaptation from north to south revealed by genomic data: guanacos from the desert to sub-antarctica**

Fabiola León^1,2,3,4^, Eduardo J. Pizarro^1,2,3,4^, Daly Noll^1,2,3,4^, Luis R. Pertierra^3^, Benito Gonzalez^5^, Warren Johnson^6^, Juan Carlos Marín^7^, Juliana A. Vianna^1,2,3,4^


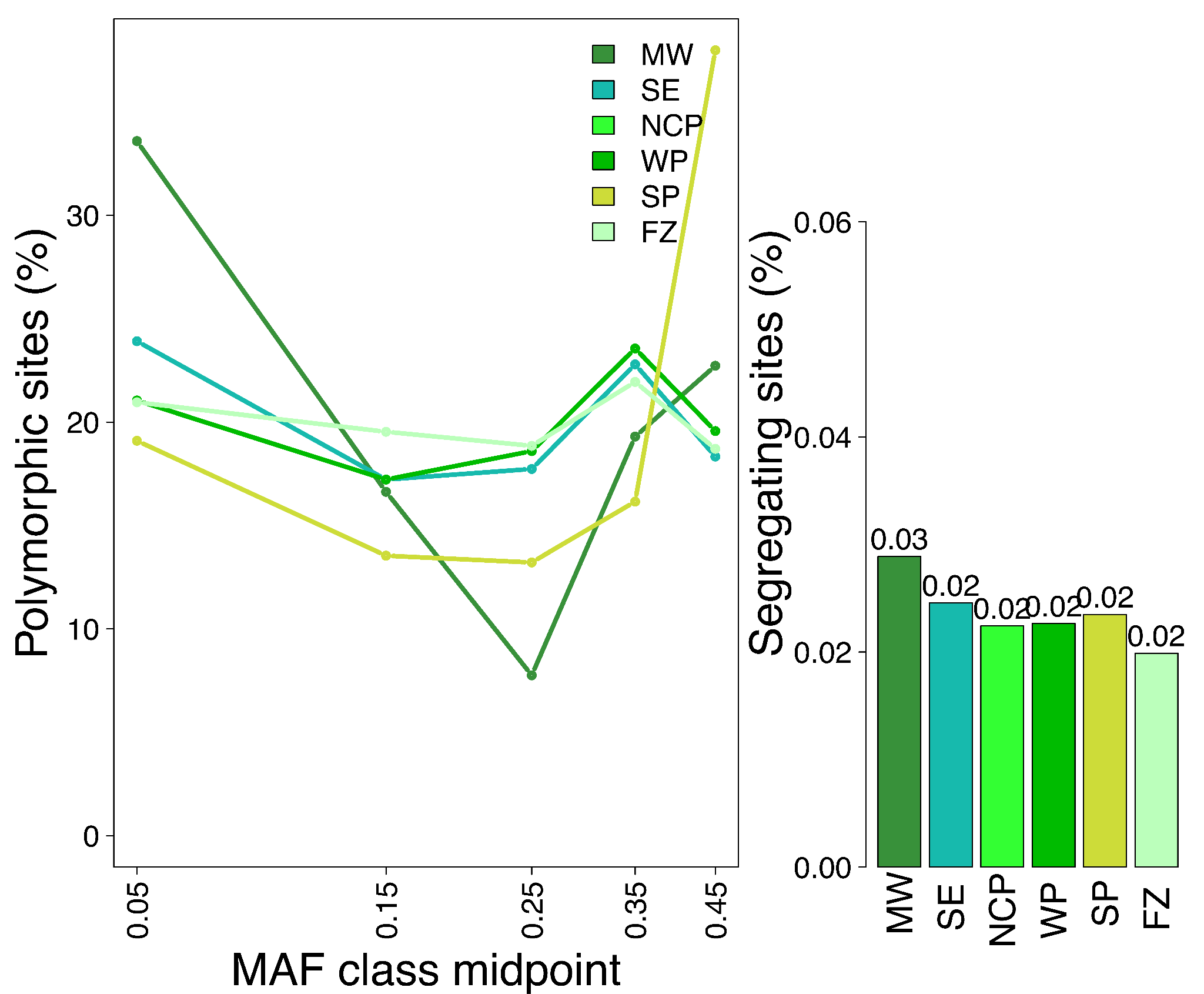


**Figure S1**. Percentage of polymorphic sites and segregating sites for the six clusters identified for *L. g. guanicoe.* The percentage of polymorphic and segregating sites indicates the proportion of these variable positions relative to the total number of sites analyzed. A higher percentage suggests greater genetic diversity within the population. The MAF midpoint likely refers to the central or median value of these frequencies across the identified clusters. It provides insights into the distribution of minor alleles within each cluster and helps characterize the allelic spectrum present in the population.


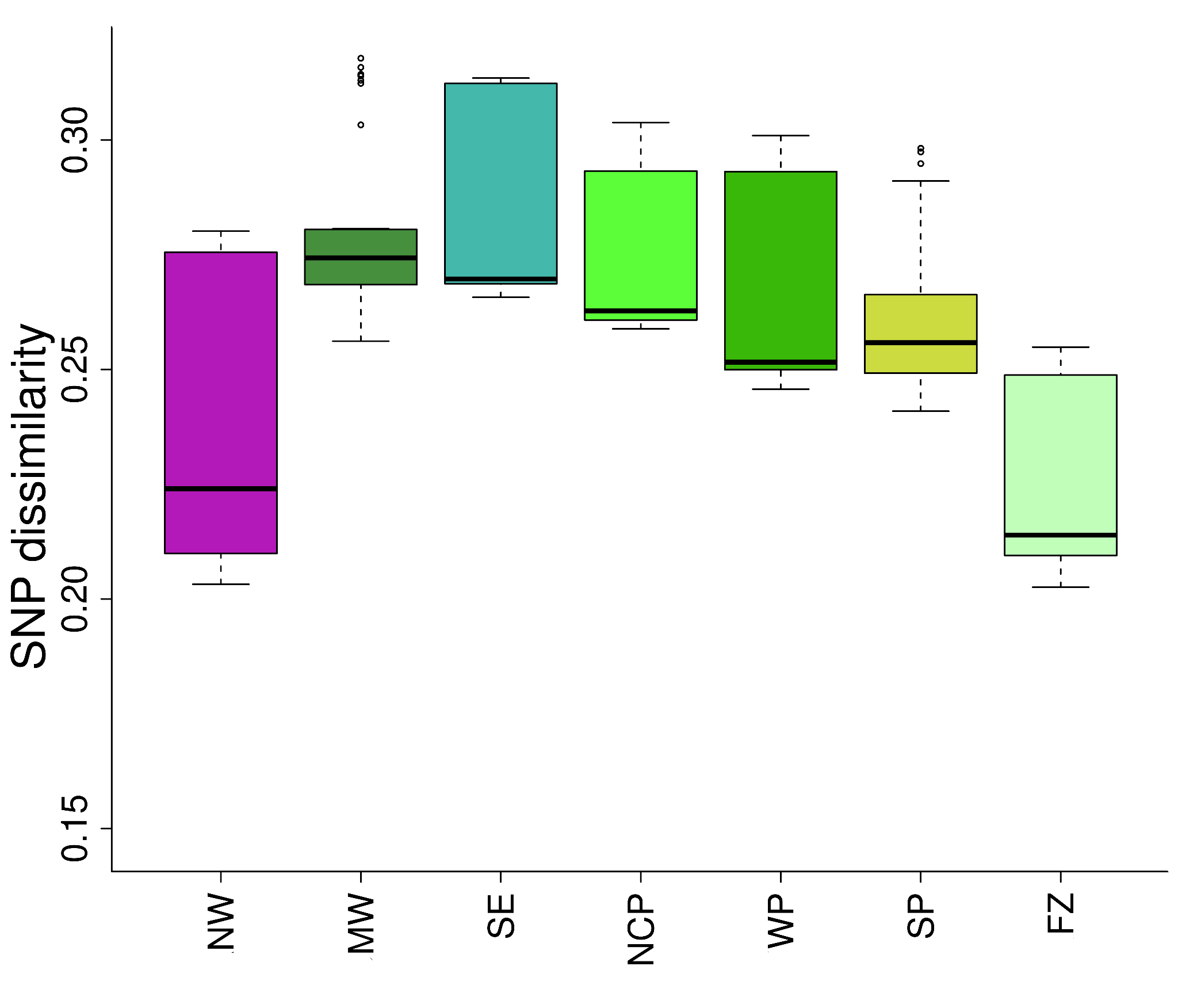


**Figure S2**. Neutral SNPs dissimilarity among genetic clusters of *L. g.guanicoe.*The dissimilarity measurement reflects the degree of genetic divergence among and within different genetic guanaco populations.


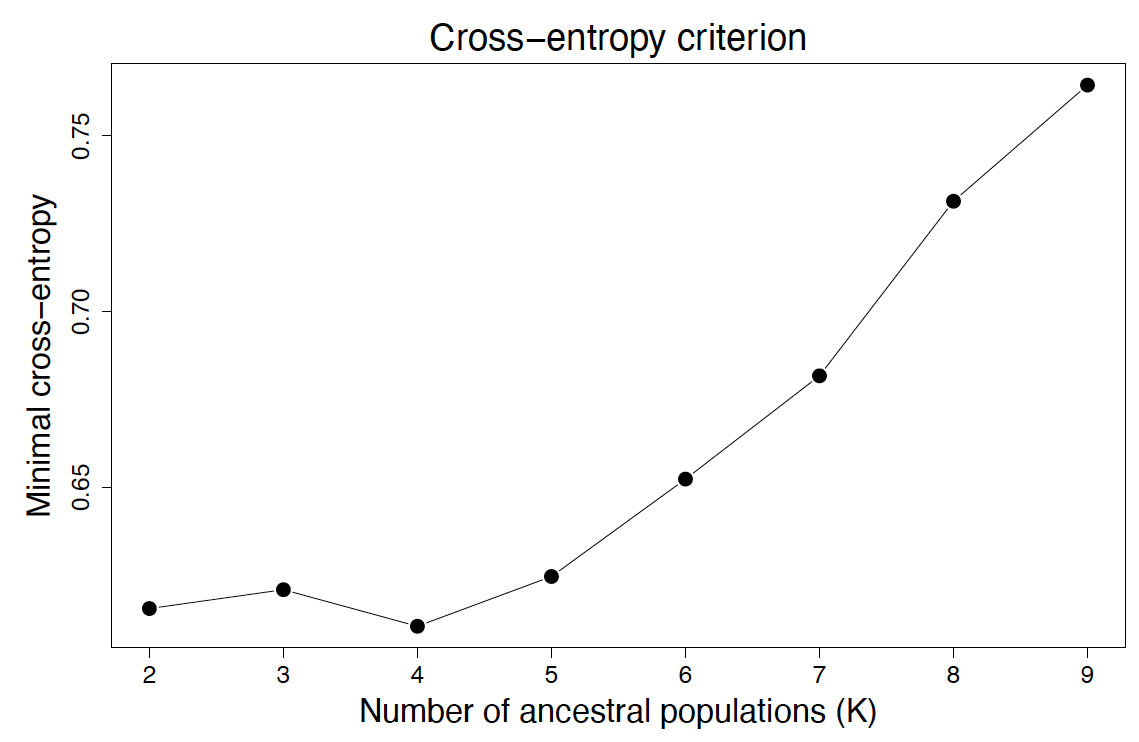


**Figure S3.** The most probable K for admixture analysis of *L. g.guanicoe.*.Based on the genetic data and the applied admixture model, the analysis suggests that the genetic structure of the population is best explained by considering four ancestral populations, and the model's predictions closely align with the observed genetic data, as indicated by a lower cross-entropy value


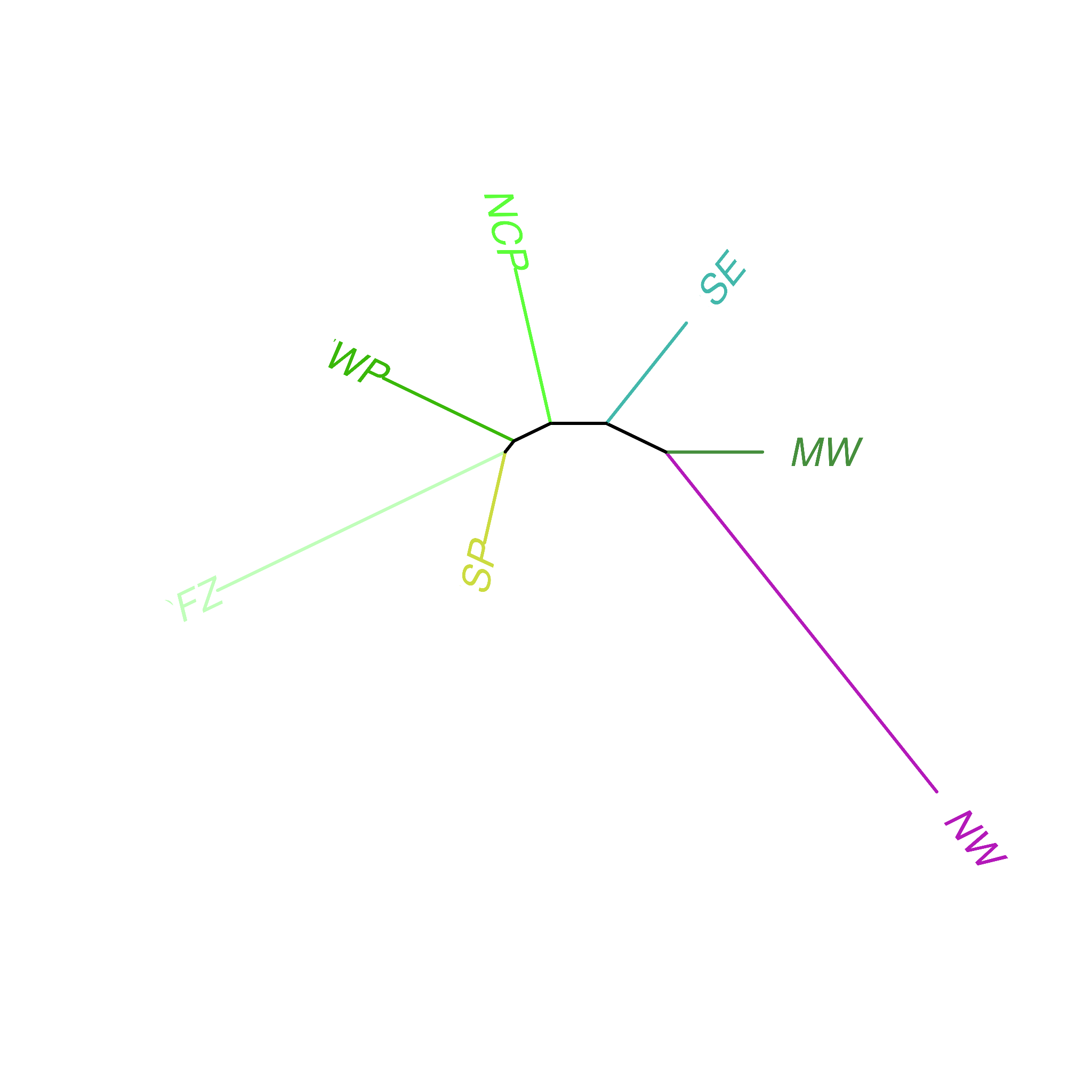


**Figure S4**. The Unrooted Neighbour Joining tree, constructed using Nei genetic distance and focusing on guanaco populations, reveals a clear pattern of genetic differentiation. The North guanaco population stands out as significantly distinct, showing a greater genetic divergence compared to the other populations, which appear more closely related to each other. This tree visually captures the genetic relationships among these populations, highlighting the notable differences between the North guanaco and the rest.


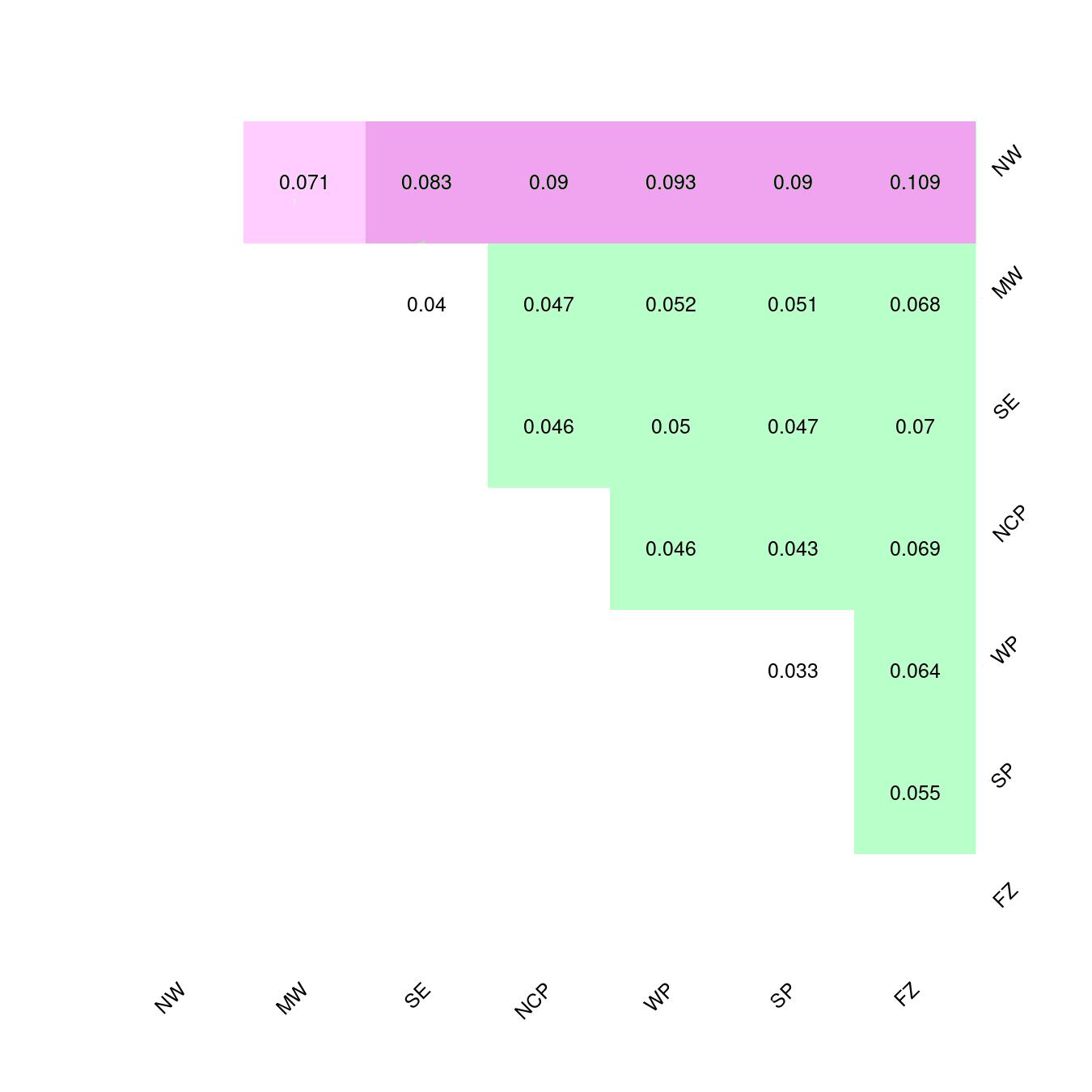


**Figure S5.** Fst index Weir and Cokherman (1984) among Guanaco populations.It quantifies the extent of genetic variation within and between populations. A high Fst value indicates significant genetic differentiation between populations, while a low value suggests genetic similarity.


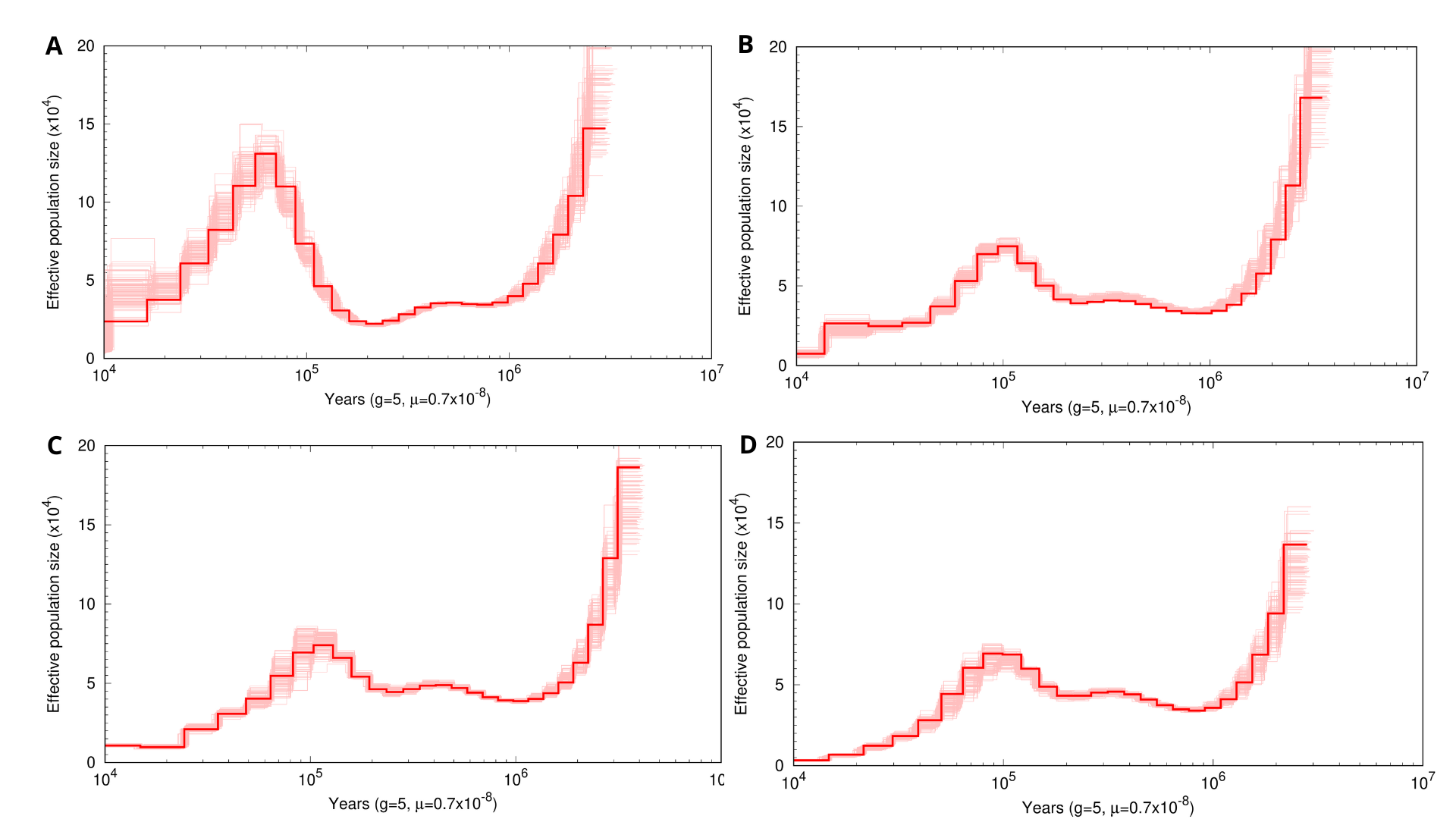


**Figure S6**. PSMC bootstrap of demographic reconstruction for guanaco, *L.g. cacsilensis* **A)** NW from Putre demonstrates a more pronounced population expansion during interglaciation periods, while **B,C** and **D** *L.g.* *guanicoe* from MW, SE and FZ displays a comparatively smaller expansion. However, both subspecies exhibit a bottleneck during the last glacial period.


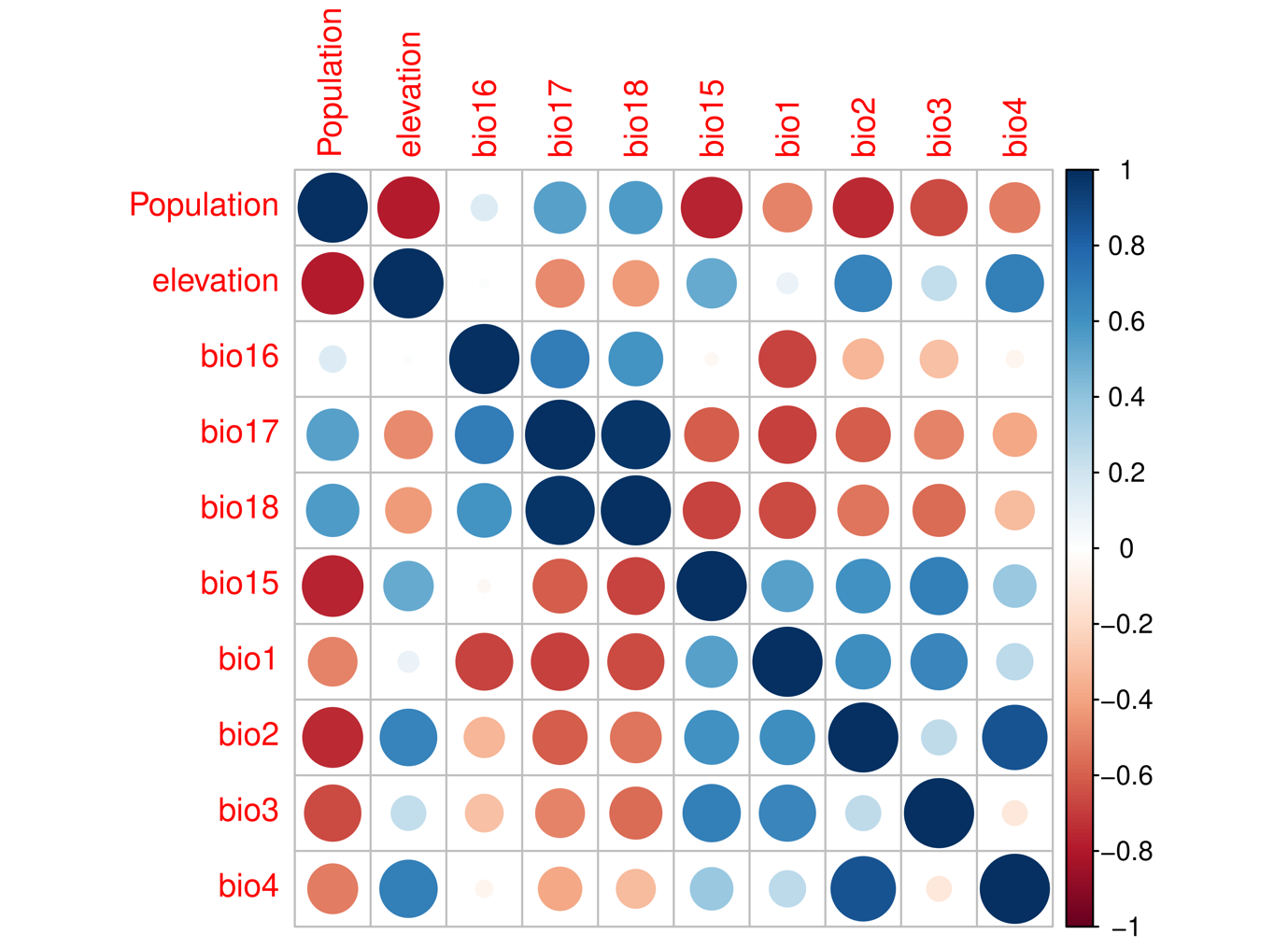


**Figure S7.** Matrix of correlation between the selected environmental variables; Population, Elevation, BIO16, BIO17, BIO18, BIO15, BIO1, BIO2, BIO3, BIO4. Values shown correspond to R-square. Size of the circles correspond to the R-square value. Colors of the circles correspond to the directionality of the correlation; positive correlation is shown in blue, while negative correlation is shown in red.


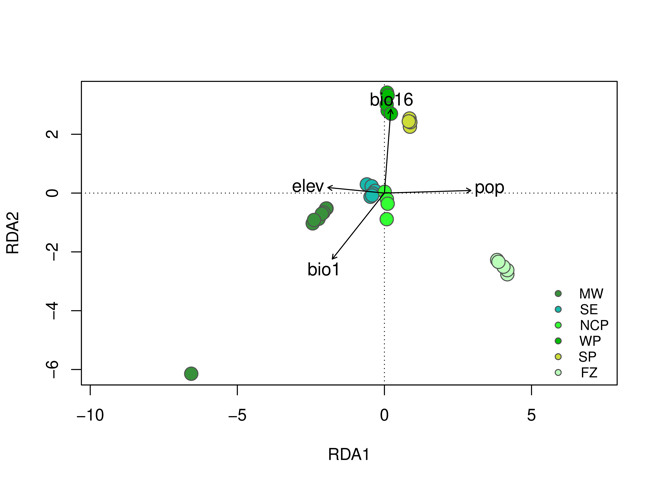

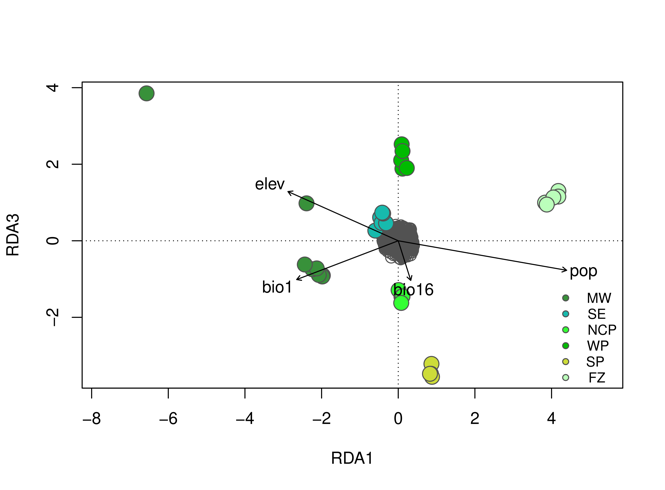


**Figure S8**. Three out of the four significant axes identified in the Redundancy Analysis (RDA) of the adaptive data for the southern guanaco *Lama guanicoe guanicoe*. This RDA includes individuals from southern populations (MW, SE, CP, WP, SP and FZ) represented by green colored circles and environmental variables depicted as black arrows.


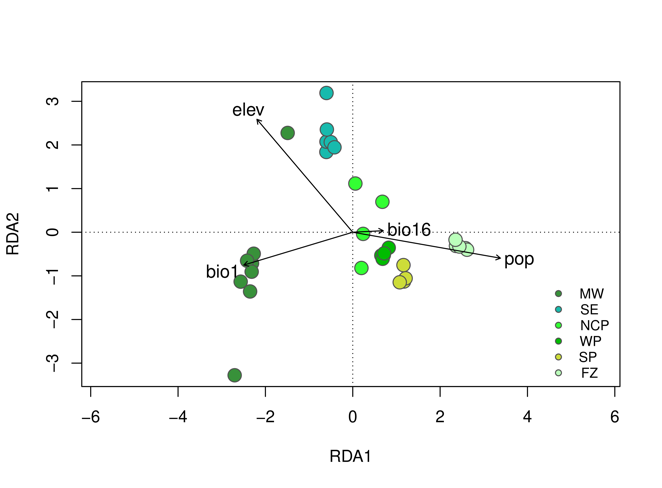

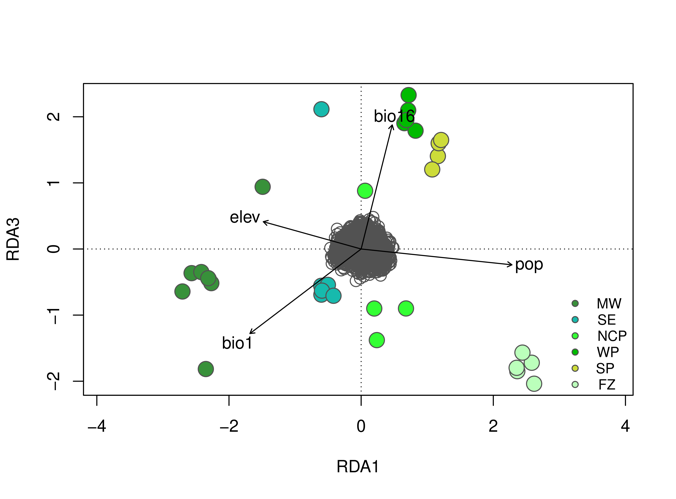


**Figure S9.** Redundancy Analysis (RDA) conducted on single nucleotide polymorphisms (SNPs) under neutral evolution within the southern guanaco population. Colored circles represent samples from various populations, potentially reflecting distinct traits or groupings. Black arrows indicate environmental variables. RDAs offer insights into the complex relationships between genomic markers, individual characteristics, and environmental influences whithin *Lama guanicoe guanicoe* subspecie


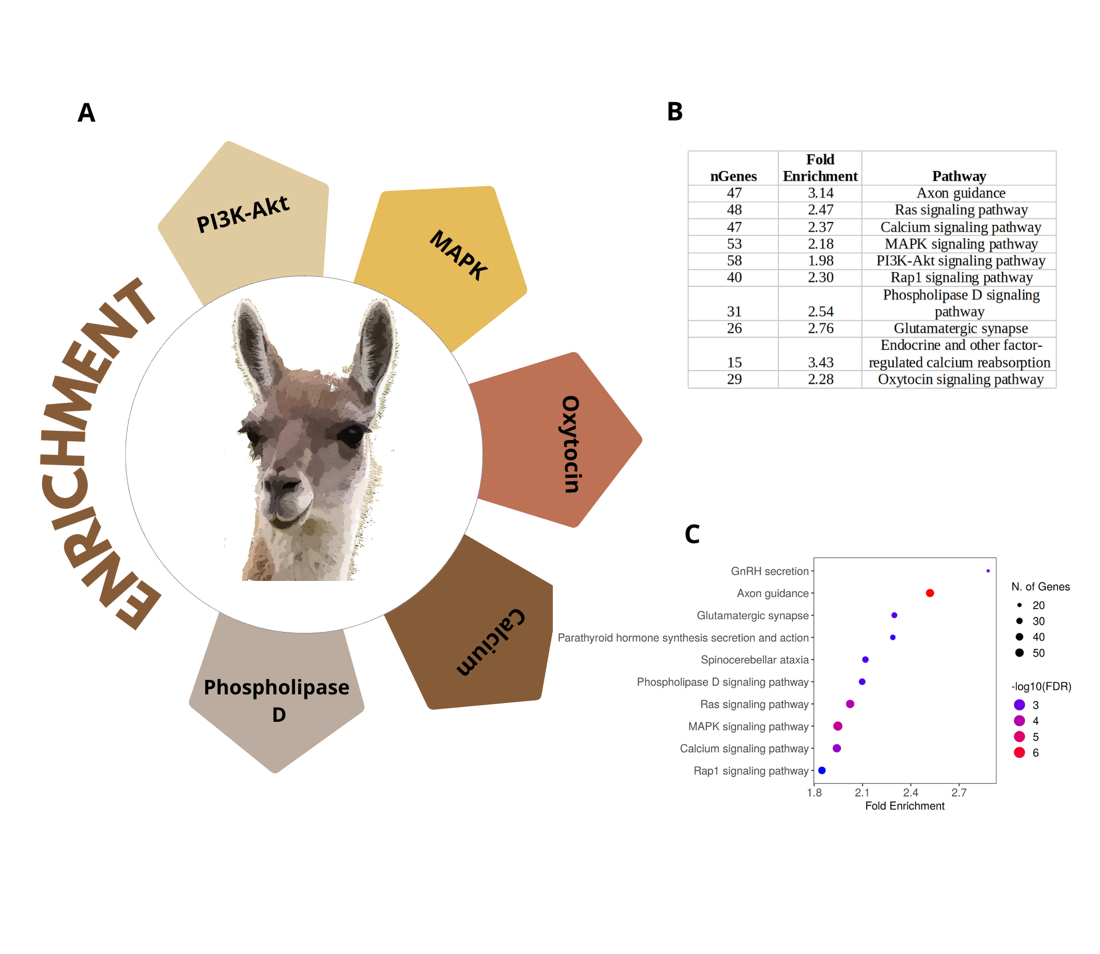


**Figure S10.** Enrichment and Gene Ontology (GO) analysis conducted on the SNPs under selection across the entire guanaco population, encompassing both subspecies. These findings elucidate enriched pathways and biological functions associated with the putative adaptative SNPs, shedding light on potential adaptive mechanisms within guanaco populations.
