# Supplementary material for: History of Diversification and Adaptation from North to South Revealed by Genomic Data: Guanacos from the Desert to Sub-Antarctica
Source: Genome Biol Evol. 2024 May 18;16(5):evae085. doi: 10.1093/gbe/evae085 (PMC11102080; doi:10.1093/gbe/evae085)
Supplement: evae085_Supplementary_Data [file evae085_supplementary_data.zip › SupplementaryMaterial_Tables_Leon.docx]

**Supplementary Material Tables**

**History of diversification and adaptation from north to south revealed by genomic data: guanacos from the desert to sub-antarctica**

Fabiola León^1,2,3,4^, Eduardo J. Pizarro^1,2,3,4^, Daly Noll^1,2,3,4^, Luis R. Pertierra^3^, Benito Gonzalez^5^, Warren Johnson^6^, Juan Carlos Marín^7^, Juliana A. Vianna^1,2,3,4^

**Table S1.** Summary of the *Lama guanicoe* samples used in the analyses, including localities (ordered roughly from north to south), grid references, type of sample (B indicates blood; M, muscle; S, skin and L, liver), number of samples used from each locality for each genetic marker. Demarcation of regions was assisted by the ML phylogeny (Figure 1B).

| Region (**Abbreviation**) | Localities, Country | Geographic position | Sample | Samples |
| --- | --- | --- | --- | --- |
|  |  | (Latitude (S); Longitude (W) | type | (N = 39) |
| Northwest (**NW**) | Huallhua, Peru | -14.833333; -74.683333 | B | 1 |
|  | Putre, Chile | -18.333333; -69.583333 | B | 4 |
|  | Paposo, Chile | -25.065156; -70.440299 | B | 1 |
| Midwest (**MW**) | Ovalle, Chile | -30.583333; -71.183333 | B | 1 |
|  | Illapel, Chile | -32.183333; -70.850000 | B | 5 |
|  | Rosin, Chile | -32.760512; -70.298611 | M | 1 |
|  | Alto Maipo, Chile | -34.084297; -70.079771 | B | 1 |
| Southeast (**SE**) | La Payunia Reserve, Argentina | -36.166667; -68.816667 | B | 5 |
| Central Patagonia (**CP**) | Somouncura Plateau, Argentina | -41.730138; -66.622777 | S | 1 |
|  | Telsen, Argentina | -42.582305; -66.538083 | S | 1 |
|  | Península Valdés, Argentina | -42.529444; -63.762083 | S | 1 |
|  | Bariloche, Argentina | -41.150000; -71.150000 | B | 1 |
|  | Monte Leon, Argentina |  | B | 1 |
| Western Patagonia (**WP**) | Valle Chacabuco, Chile | -47.600000; -72.450000 | L | 4 |
| South Patagonia (**SP**) | Torres del Paine National Park, Chile | -51.050000; -72.916667 | B | 4 |
| Fuegian zone (**FZ**) | Tierra del Fuego, Chile | -53.283333; -70.350000 | B | 5 |

**Table S2**. Significant bioclimatic variables for RDA methods.

| **VARIABLE** | **DESCRIPTION** |
| --- | --- |
| POP | Metric of population structure |
| ELEVATION | The total elevation in relation to sea level |
| BIO1 | Annual Mean Temperature |
| BIO16 | Precipitation of Wettest Quarter |

**Table S3**. Results of Redundancy Analysis (RDA) statistic significant for adaptive and neutral sets of metapopulation Single Nucleotide Polymorphisms (SNPs). The R-squared values highlight substantial explained variance, with the adaptive and the neutral set. The adjusted R-squared values account for predictor influence. Both sets exhibit significant F-statistics underscoring the importance of genetic variations in influencing the observed outcomes. Degrees of freedom are 5 for both sets, emphasizing the robustness of the statistical findings.

|  | **Rsquared** | **Rsquared Adj** | **Degree fredom**  **DF** | **Variance** | **F-statistics** | **Pr(>F)** |
| --- | --- | --- | --- | --- | --- | --- |
| **Adaptative set SNPS** | 0.4909 | 0.4272 | 4 | 3738 | 7.715 | *** |
| **Neutral set SNPS** | 0.2210 | 0.1236 | 4 | 1680.5 | 2.2701 | *** |

Signif. Codes: 0 ‘***’ 0.001 ‘**’ 0.01 ‘*’ 0.05 ‘.’ 0.1 ‘ ’ 1

**Table S4. Importance of components for adaptative SNPS data set**. Principal Component Analysis (PCA) results for RDA dimensions. Eigenvalues indicate the variance captured by each dimension. Proportion Explained shows the contribution of each dimension to the total variance explained by the model. Cumulative Proportion represents the cumulative explained variance, emphasizing that most of the variances is captured with RDA1 and with RDA3. Permutation test was performed with 999 permutations. Each axis of the model is permuted, sequentially, and calculated the Pr(>F) with the adaptive set of SNPs.

|  | **RDA1** | **RDA2** | **RDA3** |
| --- | --- | --- | --- |
| **Eigenvalue** | 2843.0955 | 507.8120 | 228.0651 |
| **Proportion Explained** | 0.7606 | 0.1359 | 0.06101 |
| **Cumulative Proportion** | 0.7606 | 0.8965 | 0.95747 |

Table S5. Component Importance for Neutral SNPs Dataset. Principal Component Analysis (PCA) for RDA dimensions. Eigenvalues signify the variance captured by each dimension. Proportion explained details the percentage of total variance per dimension. Cumulative proportion depicts the cumulative explained variance. Permutation test was performed with 999 permutations. Each axis of the model is permuted, sequentially, and calculated the Pr(>F) with the neutral set of SNPs.

|  | **RDA1** | **RDA2** | **RDA3** |
| --- | --- | --- | --- |
| **Eigenvalue** | 810.6121 | 425.4513 | 239.4329 |
| **Proportion Explained** | 0.4824 | 0.2532 | 0.1425 |
| **Cumulative Proportion** | 0.4824 | 0.7355 | 0.8780 |

**Table S6**. Results of Redundancy Analysis (RDA) statistic significant for adaptive and neutral sets of metapopulation Single Nucleotide Polymorphisms (SNPs) of the Southern subespecie (*Lama guanicoe guanicoe*). The R-squared values highlight substantial explained variance, with the adaptive and the neutral set. The adjusted R-squared values account for predictor influence. Both sets exhibit significant F-statistics underscoring the importance of genetic variations in influencing the observed outcomes. Degrees of freedom are 8 for both sets, emphasizing the robustness of the statistical findings.

|  | **Rsquared** | **Rsquared Adj** | **Degree fredom**  **DF** | **Variance** | **F-statistics** | **Pr(>F)** |
| --- | --- | --- | --- | --- | --- | --- |
| **Adaptative set SNPS** | 0.3869 | 0.2960 | 4 | 1157.3 | 4.2599 | *** |
| **Neutral set SNPS** | 0.2038 | 0.0858 | 4 | 530.97 | 1.7281 | *** |

Signif. Codes: 0 ‘***’ 0.001 ‘**’ 0.01 ‘*’ 0.05 ‘.’ 0.1 ‘ ’ 1

**Table S7. Importance of components for adaptative SNPS data set**. Principal Component Analysis (PCA) results for RDA dimensions of the Guanaco's Southern subespecie (*Lama guanicoe guanicoe*). Eigenvalues indicate the variance captured by each dimension (RDA1: 4006.9, RDA2: 474.9, RDA3: 377.7). Proportion Explained shows the contribution of each dimension to the total variance explained by the model (RDA1: 75.74%, RDA2: 8.98%, RDA3: 7.14%). Cumulative Proportion represents the cumulative explained variance, emphasizing that most of the variances is captured with RDA1 (0.7574) and with RDA3, the cumulative explained variances reach 91.86%.

|  | **RDA1** | **RDA2** | **RDA3** |
| --- | --- | --- | --- |
| **Eigenvalue** | 597.0256 | 241.0739 | 189.5231 |
| **Proportion Explained** | 0.5159 | 0.2083 | 0.1638 |
| **Cumulative Proportion** | 0.5159 | 0.7242 | 0.8880 |

Table S8. Component Importance for neutral SNPs Dataset. Results from Principal Component Analysis (PCA) for RDA dimensions of the Guanaco's Southern subespecie (*Lama guanicoe guanicoe*). Eigenvalues (RDA1: 4017.19, RDA2: 475.30, RDA3: 388.42) signify the variance captured by each dimension. Proportion Explained (RDA1: 74.39%, RDA2: 8.80%, RDA3: 7.19%) details the percentage of total variance per dimension. Cumulative Proportion (RDA1: 74.39%, RDA2: 83.19%, RDA3: 90.38%) depicts the cumulative explained variance. Notably, RDA1 emerges as pivotal, elucidating the majority of the dataset's variability.

|  | **RDA1** | **RDA2** | **RDA3** |
| --- | --- | --- | --- |
| **Eigenvalue** | 226.4681 | 111.4059 | 107.2642 |
| **Proportion Explained** | 0.4265 | 0.2098 | 0.2020 |
| **Cumulative Proportion** | 0.4265 | 0.6363 | 0.8383 |

**Table S9.** The variance inflation factors (VIF) were computed for each environmental variable incorporated into the Redundancy Analysis (RDA) focusing on putative adaptive and neutral SNPs of the southern subspecies of the Guanaco (*Lama guanicoe guanicoe*)**.**The VIF values indicate the extent to which the variance of an environmental variable is inflated due to multicollinearity with other variables in the model.

|  | **POP Structure** | **Elevation** | **BIO1** | **BIO16** |
| --- | --- | --- | --- | --- |
| **Vif** | 5.7958 | 4.1027 | 2.1822 | 3.8656 |

**Table S10:** Localization of single nucleotide polymorphisms (SNPs) under selection identified through PCAadapt analysis within coding sequences (CDS), exons, genes, messenger RNA (mRNA), and pseudogenes in the population of both subespecies of guanacos.

| **Region** | **SNPs count** |
| --- | --- |
| CDS | 420 |
| mRNA | 1869 |
| gen | 4242 |
| psudogene | 58 |
| exon | 1032 |

**Table S11.** Resecuencing basic metrics sucha as coverage and percentage of mapped reads of all individuals of guanacos against reference genome of *Camelus dromedarius.*

| **Sample** | **Population** | **Coverage** | **Maped reads of BAM files** |
| --- | --- | --- | --- |
| 1g | NW | 13,9127 | 100% |
| 3g | NW | 12,6624 | 100% |
| 4g | NW | 14,5705 | 100% |
| 5g | NW | 11,5625 | 100% |
| 6g | NW | 16,1490 | 100% |
| 7g | MW | 13,1267 | 100% |
| 8g | MW | 13,6824 | 100% |
| 9g | MW | 12,2225 | 100% |
| 10g | MW | 11,3289 | 100% |
| 11g | MW | 11,2640 | 100% |
| 12g | MW | 11,3529 | 100% |
| 13g | MW | 16,0386 | 100% |
| 14g | MW | 10,0003 | 100% |
| 15g | MW | 15,7060 | 100% |
| 16g | SE | 13,6626 | 100% |
| 17g | SE | 11,3966 | 100% |
| 18g | SE | 11,4178 | 100% |
| 19g | SE | 15,4852 | 100% |
| 20g | SE | 15,4083 | 100% |
| 21g | CP | 16,4939 | 100% |
| 22g | WP | 13,8082 | 100% |
| 23g | WP | 12,1585 | 100% |
| 24g | WP | 15,7853 | 100% |
| 25g | WP | 12,6656 | 100% |
| 26g | WP | 15,0855 | 100% |
| 27g | CP | 12,9520 | 100% |
| 28g | CP | 12,8594 | 100% |
| 29g | CP | 15,7917 | 100% |
| 30g | SP | 15,6098 | 100% |
| 31g | SP | 11,5147 | 100% |
| 32g | SP | 12,0957 | 100% |
| 33g | SP | 10,6983 | 100% |
| 36g | FZ | 14,7217 | 100% |
| 37g | FZ | 11,3428 | 100% |
| 38g | FZ | 10,7845 | 100% |
| 39g | FZ | 11,4327 | 100% |
| 40g | FZ | 11,5156 | 100% |
